# Nerve growth factor interacts with CHRM4 and promotes neuroendocrine differentiation of prostate cancer and castration resistance

**DOI:** 10.1038/s42003-020-01549-1

**Published:** 2021-01-04

**Authors:** Wei-Yu Chen, Yu-Ching Wen, Shian-Ren Lin, Hsiu-Lien Yeh, Kuo-Ching Jiang, Wei-Hao Chen, Yow-Sien Lin, Qingfu Zhang, Phui-Ly Liew, Michael Hsiao, Jiaoti Huang, Yen-Nien Liu

**Affiliations:** 1 Department of Pathology, Wan Fang Hospital, Taipei Medical University, Taipei, Taiwan; 2 Department of Pathology, School of Medicine, College of Medicine, Taipei Medical University, Taipei, Taiwan; 3 Department of Urology, Wan Fang Hospital, Taipei Medical University, Taipei, Taiwan; 4 Department of Urology, School of Medicine, College of Medicine, Taipei Medical University, Taipei, Taiwan; 5 Graduate Institute of Cancer Biology and Drug Discovery, College of Medical Science and Technology, Taipei Medical University, Taipei, Taiwan; 6 Institute of Information System and Applications, National Tsing Hua University, Hsinchu, Taiwan; 7 Biologics Development Department, Institute of Biologics, Development Center for Biotechnology, Taipei, Taiwan; 8 Department of Pathology, The First Affiliated Hospital and College of Basic Medical Sciences, China Medical University, Shenyang, China; 9 Department of Pathology, Shuang Ho Hospital, Taipei Medical University, New Taipei City, Taiwan; 10 Department of Pathology, School of Medicine, College of Medicine, Taipei Medical University, Taipei, Taiwan; 11 Genomics Research Center, Academia Sinica, Taipei, Taiwan; 12 Department of Pathology, Duke University Medical Center, Durham, NC USA

**Keywords:** Mechanisms of disease, Cancer, Cell biology, Molecular biology, Biomarkers

## Abstract

Nerve growth factor (NGF) contributes to the progression of malignancy. However, the functional role and regulatory mechanisms of NGF in the development of neuroendocrine prostate cancer (NEPC) are unclear. Here, we show that an androgen-deprivation therapy (ADT)-stimulated transcription factor, ZBTB46, upregulated *NGF* via ZBTB46 mediated-transcriptional activation of *NGF*. NGF regulates NEPC differentiation by physically interacting with a G-protein-coupled receptor, cholinergic receptor muscarinic 4 (CHRM4), after ADT. Pharmacologic NGF blockade and NGF knockdown markedly inhibited CHRM4-mediated NEPC differentiation and AKT-MYCN signaling activation. CHRM4 stimulation was associated with ADT resistance and was significantly correlated with increased NGF in high-grade and small-cell neuroendocrine prostate cancer (SCNC) patient samples. Our results reveal a role of the NGF in the development of NEPC that is linked to ZBTB46 upregulation and CHRM4 accumulation. Our study provides evidence that the NGF-CHRM4 axis has potential to be considered as a therapeutic target to impair NEPC progression.

## Abstract (web summary)

Here, the authors discover that NGF, upregulated by transcription factor ZBTB46 in prostate cancer exposed to androgen therapy, promotes neuroendocrine differentiation. They show that NGF interacts with the GPCR CHRM4, that both NGF and CHRM4 are upregulated in highly metastatic prostate cancer and that targeting NGF reduces therapy resistance in a mouse xenograft model.

## Introduction

Prostate cancer is a common health problem in men worldwide. The androgen receptor (AR) plays an important role in the development and progression of prostate cancer, due to the dependence of prostate cells on androgen for survival and growth^1^. However, prostate cancer is often heterogeneous and shows different sensitivities to androgen-deprivation therapy (ADT) based on tumor grades (or Gleason patterns). Most prostate cancer patients who undergo ADT develop drug-resistant disease called castration-resistant prostate cancer (CRPC)^2^. With prolonged ADT, some prostate tumors transform into carcinomas with neuroendocrine differentiation termed neuroendocrine prostate cancer (NEPC) or small-cell neuroendocrine prostate cancer (SCNC), which demonstrates a loss of AR signaling and aggressive androgen-independent phenotypes^3^. The molecular effectors driving NEPC or SCNC differentiation in this background are largely unknown, as biopsies are rarely performed on these patients^4,5^. Thus, biomarkers are critically needed to stratify biopsy-diagnosed NEPC so that patients with aggressive NEPC can be offered appropriate treatment.

The nerve growth factor (NGF) functions as a regulator of neuronal function and modulates its responses via the tyrosine kinase receptor, NTRK1, and the neurotrophin receptor, NGF receptor (NGFR)^6^. The NGF was reported to be extensively associated with the development of neuronal tissues, is known to participate in angiogenesis, and upregulates expressions of oncogenes in several tissues^7^. Indeed, involvement of the NGF in pancreatic cancer was demonstrated to increase cell proliferation and survival through activation of mitogen-activated protein kinase (MAPK) via the NTRK1 receptor^8^. In breast cancer, the NGF is known to mediate an antiapoptotic effect by activating nuclear factor-κB via the NGFR^9^. Thus, the NGF plays dual roles by stimulating the two receptors via separate signaling pathways^10^. The NGF was reported to promote prostate cancer cell metastasis^11,12^, yet the mechanisms and functions of NGF in NEPC differentiation have not been clearly elucidated. As NEPC tumors display AR-null phenotypes and contain neurotrophic factors^13^, it is important to understand the role of the NGF of mediating androgen-independent signaling in the viability of these cells.

The muscarinic acetylcholine receptor (mAChR, also known as chlorogenic receptor muscarinic, CHRM) is a subclass of acetylcholine receptors which belongs to G-protein-coupled receptors (GPCRs), and was shown to be involved in the transduction of cholinergic signals in the central nervous system^14^. Activated cholinergic signaling was demonstrated to be directly relevant to the progression of cancer in colorectal, small-cell lung cancer (SCLC), gastric, and pancreatic tumorigenesis^15–17^. In prostate cancer, CHRM3 activation was reported to promote cell proliferation in vitro^18^. Autonomic nerve system-mediated prostate cancer progression occurs through activation of CHRM1 signaling^19,20^. CHRM4 is activated in neuronal signaling by interacting with acetylcholine and conjugated secondary bile acids^21^; however, the role of CHRM4 in prostate cancer progression remains unidentified. In the microenvironment of prostate cancer that underwent ADT resistance, whether neurotrophic factors or neuropeptides secreted by prostate neuroendocrine-like cells play a role in promoting NEPC progression of non-neuroendocrine cells via activation of a muscarinic receptor has not yet been studied. We sought to study the communication mechanism between the NGF and CHRM4 to provide an exploitable target for effective NEPC treatment and diagnostic strategies.

Recent studies suggested that anti-androgen therapy contributes to androgen independence, which may result in NEPC^22^. Our earlier study demonstrated that ADT activates ZBTB46, a prostatic tumor promoter, which promotes the epithelial–mesenchymal transition through transcriptional regulation of *SNAI1*^23^ and is associated with neuroendocrine differentiation and tumor recurrence in prostate cancer after ADT^24^. Herein, we present evidence that ZBTB46 regulates activation of the NGF, thereby facilitating the development of NEPC and drug-resistant phenotypes. We identified consensus molecular pathways in prostate cancer cells that are modulated by the NGF–CHRM4-upregulated neuroendocrine-like phenotype via activation of the ZBTB46 transcription factor. The results suggest an approach for NEPC treatment by targeting NGF–CHRM4 signaling.

## Results

### ZBTB46-upregulated NGF is associated with NEPC differentiation

**Fig. 1:**
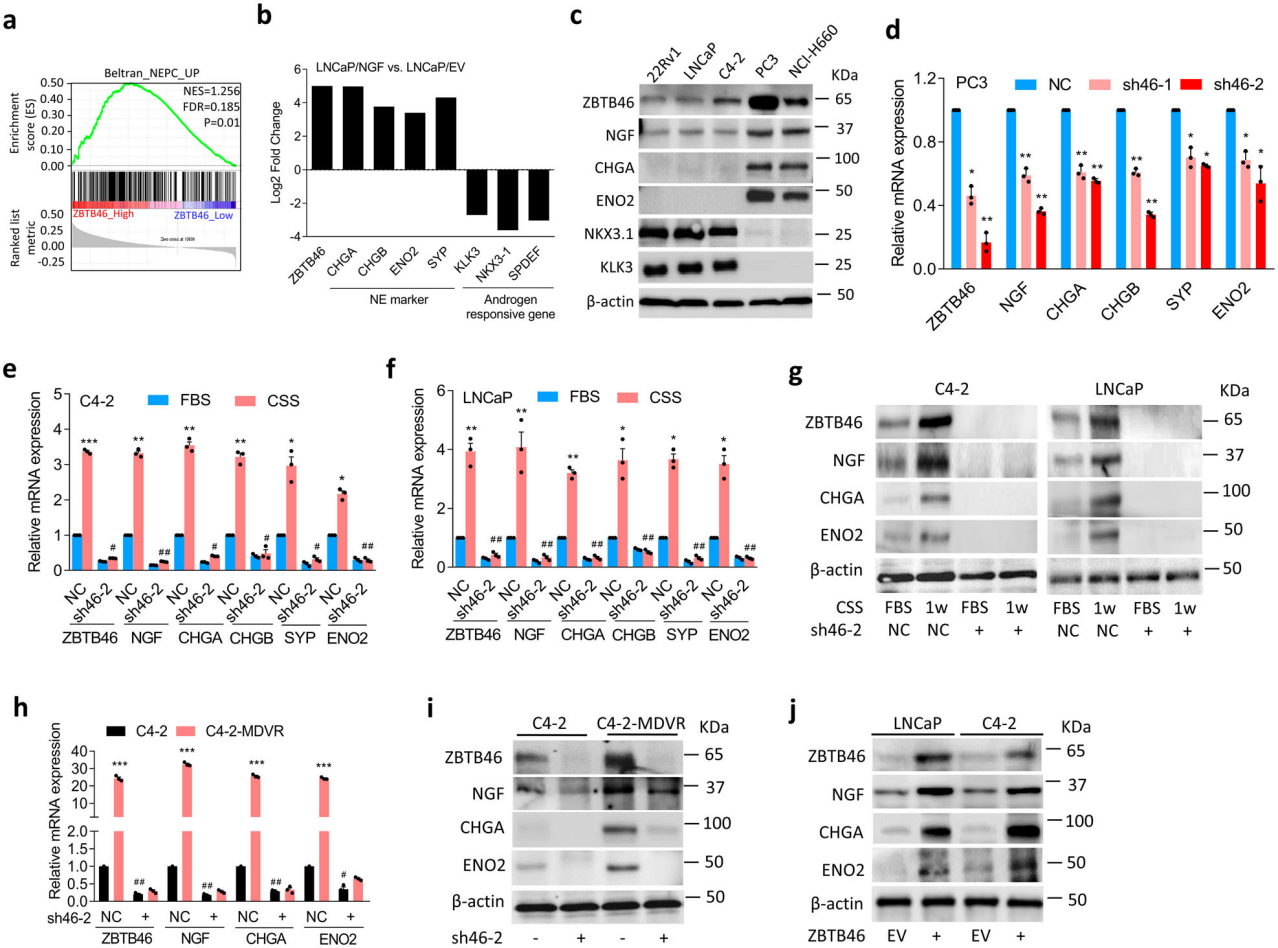
ADT-upregulated ZBTB46 is associated with the NGF and promotes neuroendocrine differentiation of prostate cancer. **a** GSEA of TCGA prostate cancer dataset showed that higher ZBTB46 expression of prostate tissues was positively associated with NEPC-responsive gene signature. NES normalized enrichment score, FDR false discovery rate. **b** Relative expression of ZBTB46, neuroendocrine markers, and androgen-responsive genes determined by RNA-Seq analysis with LNCaP cells expressing the NGF vs. LNCaP cells expressing an empty vector (EV). The relative expression was determined by the ratio of LNCaP-NGF/LNCaP-EV. **c** Western blotting of ZBTB46, NGF, CHGA, ENO2, NKX3-1, and KLK3 in various prostate cancer cells. **d** Relative mRNA levels of ZBTB46, NGF, CHGA, CHGB, SYP, and ENO2 in PC3 cells with stable expression of the non-target control (NC) or ZBTB46 shRNA vector. **e**, **f** ZBTB46, NGF, CHGA, CHGB, SYP, and ENO2 mRNA levels in NC and ZBTB46-knockdown C4-2 (**e**) and LNCaP (**f**) cells cultured with 10% charcoal stripped serum (CSS)-containing medium for 1 week. * vs. Fetal bovine serum (FBS); ^#^ vs. the NC. **g** Western blotting of ZBTB46, NGF, CHGA, and ENO2 in ZBTB46-knockdown C4-2 and LNCaP cells treated with CSS-containing medium for 1 week. **h**, **i** ZBTB46, NGF, CHGA, and ENO2 mRNA (**h**) and protein (**i**) levels in C4-2 and MDV3100-resistant C4-2 (C4-2-MDVR) cells following ZBTB46-knockdown. * C4-2 vs. C4-2-MDVR; ^#^ vs. the NC. Data from the quantification of mRNA are presented as the mean ± SEM of three independent experiments; *n* = 3 per group. *p < 0.05, **p < 0.01, ***p < 0.001; by a two-way ANOVA. **j** ZBTB46, NGF, CHGA, and ENO2 protein levels in LNCaP and C4-2 cells stably expressing an EV or a ZBTB46 cDNA expression vector.

We previously reported that the ADT-activated transcription factor, ZBTB46, is associated with drug resistance and metastasis of prostate cancer^23^. As to relationships among ZBTB46, AR signaling, and NEPC progression, we checked expression levels of ZBTB46, neuroendocrine markers (chromogranin A/B (*CHGA/B*) and enolase 2 (*ENO2*)), and androgen-responsive genes (kallikrein-3 (*KLK3*) and NK3 homeobox 1 (*NKX3-1*)) in an RNA-sequence (RNA-Seq) dataset (GSE48403) in paired prostate cancer samples pre-ADT and post-ADT. We found that patients post-ADT had increased ZBTB46 and neuroendocrine marker levels but had decreased androgen-responsive gene expressions in this dataset (Supplementary Fig. 1a). To study the regulatory mechanisms involved in ZBTB46 upregulation during NEPC development, we examined relationships between ZBTB46 and activation of signatures of NEPC-responsive genes in The Cancer Genome Atlas (TCGA) prostate cancer dataset. Results showed that tissues expressing high levels of ZBTB46 were associated with an upregulated NEPC-responsive signature in the prostate cancer dataset by a gene set enrichment analysis (GSEA)^25^ (Fig. 1a). Interestingly, *NGF* was found to be a candidate gene among the top group in the ranked gene list of NEPC-responsive genes (Supplementary Fig. 1b). GSEA analyses from TCGA prostate cancer database confirmed that patients with higher NGF levels were positively associated with gene signatures associated with upregulated neuronal developmental-responsive signaling (KEGG, Gene Ontology, and Reactome, Supplementary Fig. 1c). To determine whether the NGF is important for NEPC differentiation, messenger (m)RNA from LNCaP cells stably expressing the NGF or a control vector was prepared and used in an RNA-Seq analysis. mRNA levels of neuroendocrine markers and androgen-responsive genes were comparable. We found that NGF overexpression was positively associated with ZBTB46 and neuroendocrine markers, and was negatively associated with androgen-responsive genes (Fig. 1b). We measured ZBTB46 and NGF expressions in a panel of prostate cancer cell lines, and we found that AR-negative PC3^26^ cells and NEPC-like NCI-H660^5^ cells had higher ZBTB46 and NGF expressions and were positively associated with neuroendocrine marker expressions compared to AR-positive 22Rv1, LNCaP, and C4-2 cells (Fig. 1c). In addition, decreases in androgen-responsive genes (*KLK3* and *NKX3-1*) were observed in PC3 and NCI-H660 cells compared to AR-positive cells (Fig. 1c). To analyze whether NGF expression is regulated by ZBTB46 and is associated with NEPC differentiation, we stably introduced ZBTB46 short hairpin (sh)RNA into PC3 cells. We found that cells with ZBTB46-knockdown exhibited significantly decreased NGF and neuroendocrine marker expressions (sh46-1 and sh46-2, Fig. 1d). Furthermore, increased NGF mRNA levels were found to be dependent on ZBTB46, as ZBTB46-knockdown reduced NGF and neuroendocrine marker expressions in C4-2 and LNCaP cells, regardless of an ADT-mimicking condition (achieved using charcoal-stripped serum (CSS)-containing medium) (Fig. 1e, f). NGF protein expression was shown to be associated with ZBTB46 and neuroendocrine markers as confirmed by Western blotting in cells harboring ZBTB46-knockdown after ADT (Fig. 1g). We further tested whether expression of the NGF is regulated by suppression of AR signaling. Indeed, long-term treatment of AR-positive C4-2 cells with the AR antagonist, enzalutamide (MDV3100), produced increased ZBTB46, NGF, and neuroendocrine marker mRNA and protein expressions, whereas ZBTB46-knockdown abolished those alterations (Fig. 1h, i). Conversely, upregulation of the NGF and neuroendocrine markers was confirmed to be positively associated with ZBTB46 overexpression in LNCaP and C4-2 cells (Fig. 1j). These results indicate that ADT-increased NGF promotes NEPC differentiation and suggest that NGF expression is likely regulated by ZBTB46.

### ZBTB46 directly binds to the regulatory sequence of the *NGF* and upregulates NGF expression

**Fig. 2:**
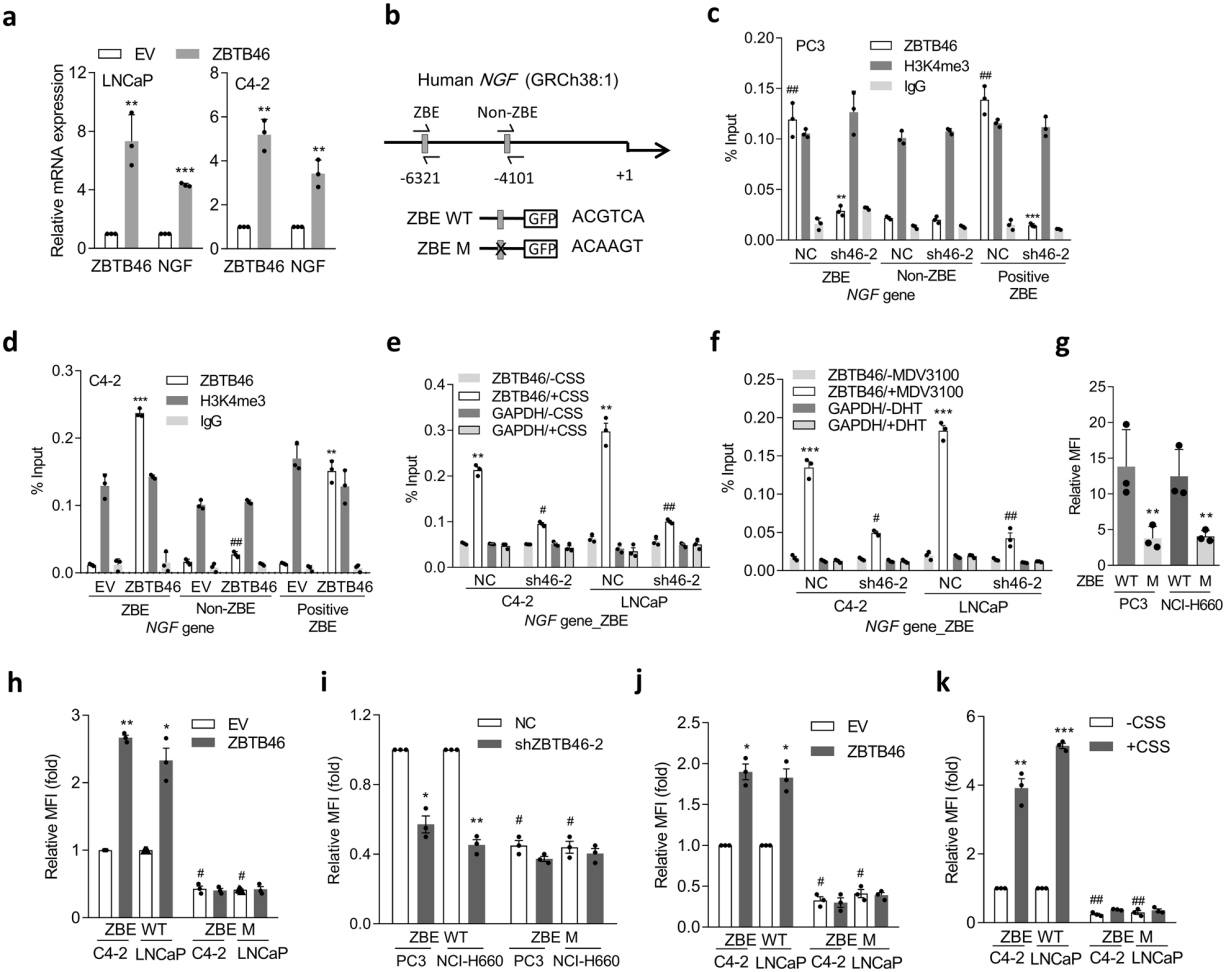
The NGF is regulated by ZBTB46 in prostate cancer cells. **a** ZBTB46 and NGF mRNA levels in LNCaP and C4-2 cells stably expressing an empty vector (EV) or a ZBTB46 cDNA expression vector. * vs. the EV. Data from the quantification of mRNA are presented as the mean ± SEM of three independent experiments; *n* = 3 per group. ** *p* < 0.01, *** *p* < 0.001; by a two-way ANOVA. **b** Schematic of the predicted ZBTB46-binding element (ZBE) or a non-ZBE and the introduced binding site mutant in the regulatory sequence reporter constructs of human *NGF*. **c** ChIP assays of PC3 **c**ells following ZBTB46-knockdown (sh46-2) using a specific antibody against ZBTB46 for IP; NC non-target control. Antibodies against H3K4me3 and IgG, respectively, served as positive and negative controls. Precipitated DNA was quantified via a qPCR of ZBE, non-ZBE, and positive ZBE sites. Enrichment is given as a percentage of the total input. * vs. the NC; ^#^ vs. the ZBE. **d** ChIP assays in C4-2 cells following overexpression of ZBTB46. * vs. the EV; ^#^ vs. the ZBE. **e**, **f** ChIP assays of C4-2 and LNCaP cells following ZBTB46-knockdown and treatment with charcoal stripped serum (CSS)-containing medium (**e**) or 10 μM MDV3100 (**f**) for 48 h. * −CSS vs. +CSS; ^#^ vs. the NC. Data from the ChIP assays are presented as the mean ± SEM of three independent experiments; *n* = 3 per group. **p < 0.01, ***p < 0.001; by a two-way ANOVA. **g** Relative median fluorescence intensity (MFI) of the wild-type (WT) and mutant (M) *NGF* reporters in PC3 and NCI-H660 cells. * vs. the WT. **h, i** WT and M *NGF* reporter activities in response to ZBTB46 overexpression in C4-2 and LNCaP cells (**h**) or ZBTB46-knockdown in PC3 and NCI-H660 cells (**i**). * vs. the EV or NC; ^#^ vs. the ZBE WT. **j**, **k** Relative MFI of the WT and M *NGF* reporters in C4-2 and LNCaP cells after treatment with 10 μM MDV3100 (**j**) and CSS-containing medium (**k**) for 48 h. * vs. DMSO or −CSS; ^#^ vs. the ZBE WT. Data from relative MFIs are presented as the mean ± SEM of three independent experiments. * *p* < 0.05, ** *p* < 0.01, *** *p* < 0.001; by a two-way ANOVA.

We hypothesized that ZBTB46 upregulates NGF expression in prostate cancer cells by acting as a transcriptional activator and binding to a ZBTB46-binding element (ZBE) in the *NGF* regulatory sequence. Significantly, after expressing a ZBTB46-complementary (c)DNA vector in AR-positive cells, we observed an increase in the NGF mRNA level (Fig. 2a). We searched for sequences resembling the ZBE in the putative *NGF* regulatory sequence region and found a candidate ZBE at nucleotide −6321 relative to the *NGF* transcriptional start site (Fig. 2b). We performed chromatin immunoprecipitation (ChIP) assays and observed significantly high ZBTB46 binding at the putative ZBE compared to a non-ZBTB46-binding site (non-ZBE; Fig. 2c). A positive control ZBE (positive ZBE) *SNAI1* promoter^23^ was used as a control; this element also showed significantly high ZBTB46 binding (Fig. 2c). We observed a decrease in ZBTB46-binding activity at the ZBE and a positive ZBE in the presence of ZBTB46 short hairpin (sh)RNA in AR-negative PC3 cells (sh46-2, Fig. 2c). Conversely, ZBTB46 overexpression in AR-positive C4-2 cells showed increased ZBTB46-binding activity (Fig. 2d). Moreover, ZBTB46-binding signals were enriched in C4-2 and LNCaP cells in response to CSS-containing medium or MDV3100 (Fig. 2e, f), supporting the hypothesis that ADT-increased ZBTB46 upregulates *NGF* expression. We also found that even in cells treated with CSS-containing medium or MDV3100, the ZBTB46-binding signal in ZBTB46-knockdown cells decreased (Fig. 2e, f), supporting direct interaction between ZBTB46 and *NGF* after ADT. Next, we performed reporter assays using two DNA constructs, one containing an individual wild-type (WT) ZBE (ZBE WT) and another containing a mutant ZBE (ZBE M) from the *NGF-*regulatory sequence (Fig. 2b). Compared to ZBE WT, ZBE M showed decreased reporter activity when these constructs were transfected into PC3 and NCI-H660 cells (Fig. 2g). We also found that the ZBE M abolished the ability of ZBTB46 overexpression (Fig. 2h) and ZBTB46-knockdown (Fig. 2i) increased or decreased the reporter activity in AR-positive or AR-negative cells, respectively. Moreover, MDV3100-containing or CSS-containing medium-treated AR-positive cells showed significantly increased reporter activity relative to untreated cells, whereas the ZBE M exhibited disruption of their upregulated reporter activity (Fig. 2j, k). These findings suggest a mechanism whereby ADT-upregulated ZBTB46 enhances *NGF* transcription through direct physical interaction with the *NGF*-regulatory sequence.

### Repression of AR signaling increases the NGF and is associated with NEPC differentiation

**Fig. 3:**
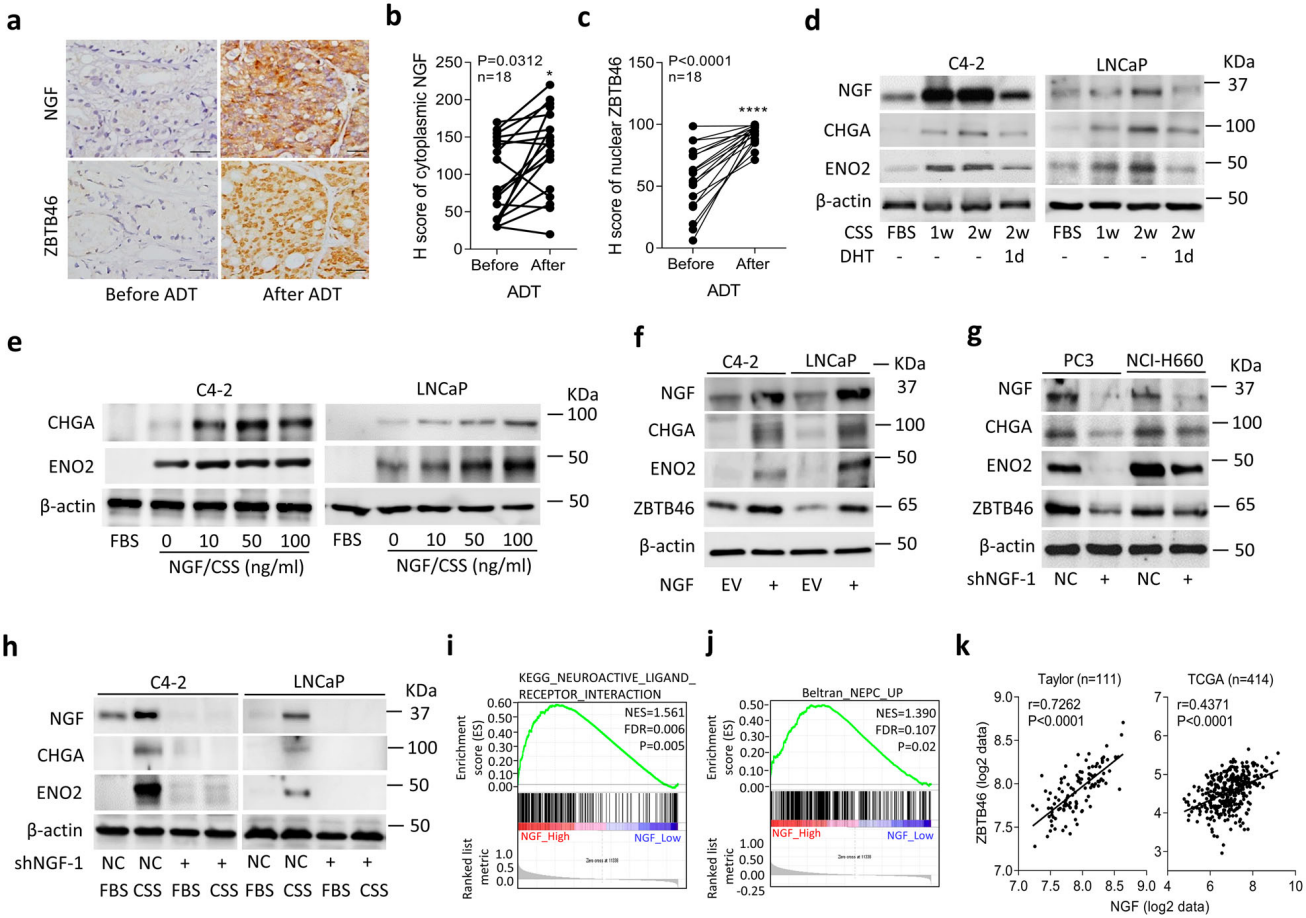
ADT-upregulated NGF is associated with NEPC. **a–c** IHC staining (**a**) and analysis (**b** and **c**) of cytoplasmic NGF and nuclear ZBTB46 in prostate cancer tissue sections from patients before and after ADT. The 18 samples were collected from Taipei Medical University-Wan Fang Hospital. Scale bars, 100 µm. Statistical analysis was performed using a two-tailed Student’s *t*-test. *p < 0.05, ****p < 0.0001. **d** Western blotting of the NGF, CHGA, and ENO2 in C4-2 and LNCaP cells cultured in charcoal stripped serum (CSS)-containing medium for 1 and 2 weeks, followed by treatment with 10 nM dihydrotestosterone (DHT) for 1 day. **e** Western blotting for CHGA and ENO2 in C4-2 and LNCaP cells treated with the NGF protein in CSS-containing medium at various concentrations for 1 week. Fetal bovine serum (FBS)-containing medium without the NGF protein served as the control. **f** Western blotting for NGF, CHGA, ENO2, and ZBTB46 in C4-2 and LNCaP cells stably transfected with an empty vector (EV) or NGF expression vector. **g** Western blotting for NGF, CHGA, ENO2, and ZBTB46 in PC3 and NCI-H660 cells stably expressing a non-target control (NC) or NGF shRNA vector. **h** Protein levels of the NGF, CHGA, and ENO2 in NGF-knockdown C4-2 or LNCaP cells and cells treated with CSS-containing medium for 1 week. **i**, **j** GSEAs of TCGA prostate cancer dataset showing that high NGF expression levels in prostate tissues were positively associated with a neuronal development signature (KEGG) (**i**) and a NEPC-respons**i**ve gene signature^25^ (**j**). NES normalized enrichment score, FDR false discovery rate. **k** Correlation analysis of *NGF* with ZBTB46 mRNA levels in clinical tissue samples from the Taylor and TCGA prostate cancer datasets. Significance was determined by a two-way ANOVA.

To investigate whether NGF stimulation is associated with ZBTB46 after ADT, we examined prostate cancer samples consisting of tissue specimens collected from 18 prostate cancer patients before and after ADT at Taipei Medical University-Wan Fang Hospital (Taipei, Taiwan). Immunohistochemical (IHC) staining revealed that significantly higher cytoplasmic NGF levels were associated with nuclear ZBTB46 expression in paired samples from the same patients after ADT (Fig. 3a–c). To measure NGF expression in AR-positive cells relative to the AR signaling response, we cultured AR-positive C4-2 and LNCaP cells in CSS-containing medium to mimic an androgen-deprivation condition. We found that CSS-treated cells had increased levels of the NGF and were associated with higher neuroendocrine marker expressions compared to cells cultured in fetal bovine serum (FBS)-containing medium; however, AR ligand (dihydrotestosterone (DHT))-treated cells had reduced levels of NGF and neuroendocrine markers (Fig. 3d; Supplementary Fig. 2a). We further examined NGF expression with signatures reflecting AR signaling components in TCGA prostate cancer dataset and found that tissues expressing low levels of the NGF were significantly associated with gene signatures of upregulated androgen responsiveness^27,28^ by a GSEA (Supplementary Fig. 2b). In contrast, high NGF levels were associated with a downregulated androgen-responsive signature^29^ according to the GSEA (Supplementary Fig. 2c). These findings support the notion that ADT upregulates NGF expression and is involved in neuroendocrine differentiation of prostate cancer cells.

Although the NGF was shown to be involved in neuronal development^7^, we extended our analysis to the contribution of the NGF to neuroendocrine differentiation in prostate adenocarcinomas. We cultured LNCaP and C4-2 cells in CSS-containing medium to mimic ADT and further treated those cells with the NGF for 7 days. neuroendocrine markers were detected in CSS-containing medium without NGF treatment, since we expected ADT to stimulate the neuroendocrine phenotype, and more neuroendocrine markers were expressed in CSS-containing medium-treated cells in the presence of the NGF (Fig. 3e). Exogenous NGF strengthened the increase in neuroendocrine markers in an ADT condition, suggesting that the NGF might facilitate AR signaling inhibition-driven NEPC differentiation. Notably, these changes were confirmed to be positively associated with NGF cDNA vector overexpression in LNCaP and C4-2 cells, which showed increased neuroendocrine marker expressions (Fig. 3f; Supplementary Fig. 2d). Conversely, we found that NGF-knockdown in PC3 and NCI-H660 cells was associated with decreased neuroendocrine marker expressions (Fig. 3g; Supplementary Fig. 2e). Immunofluorescence staining was carried out in LNCaP and C4-2 cells stably expressing an empty vector (EV) or NGF cDNA vector using NGF and ENO2 antibodies. Cells with NGF overexpression had increased NGF and ENO2 expressions, supporting NGF-promoting NEPC differentiation (Supplementary Fig. 2f). We also found that NGF overexpression or NGF-knockdown in prostate cancer cells, respectively, increased or decreased ZBTB46 expression (Fig. 3f, g), suggesting that activated NGF may play a positive feedback role in regulating ZBTB46. Moreover, AR-positive cells subjected to CSS-containing medium treatment had higher NGF and neuroendocrine marker expressions compared to cells treated with FBS-containing medium (Fig. 3h). However, CSS-containing medium treatment did not increase levels of NGF or neuroendocrine markers in NGF-knockdown cells (Fig. 3h). Moreover, cells with AR-knockdown showed increased NGF and neuroendocrine marker expressions, and overexpression of NGF synergistically increased those markers (Supplementary Fig. 2g). These data support AR inhibition possibly upregulating the NGF, by which it promotes ADT-driven NEPC differentiation. Next, we overexpressed the NGF and knocked-down ZBTB46 in C4-2 and LNCaP cells to clarify whether ZBTB46 promotes NEPC progression through NGF. NGF overexpression increased neuroendocrine marker expressions; however, neuroendocrine marker expressions in NGF-transfected cells with ZBTB46-knockdown did not increase as much as those of the non-target control (NC)-transfected cell group (Supplementary Fig. 3a, b). These data suggest that the NGF may be one of the targets of the ZBTB46 transcription factor that regulates NEPC differentiation. We also observed that NC-transfected cells with NGF overexpression had increased ZBTB46 expression (Supplementary Fig. 3a, b), supporting the NGF possibly regulating ZBTB46 expression through positive feedback. The GSEA validated that prostate cancer tissues expressing higher NGF levels were involved in neuronal development (Fig. 3i) and in response to upregulated NEPC signatures^25^ (Fig. 3j) in TCGA prostate cancer dataset. Moreover, mean expression correlations were examined in the Taylor^30^ and TCGA prostate cancer datasets, showing that the NGF was positively correlated with ZBTB46 and neuroendocrine marker expressions and inversely correlated with androgen-responsive gene expressions according to a correlation analysis (Fig. 3k; Supplementary Fig. 3c). These results indicated that upregulation of the NGF enhances neuroendocrine differentiation in prostate cancer after ADT.

### Targeting the NGF reduces ADT resistance and neuroendocrine differentiation of prostate cancer

**Fig. 4:**
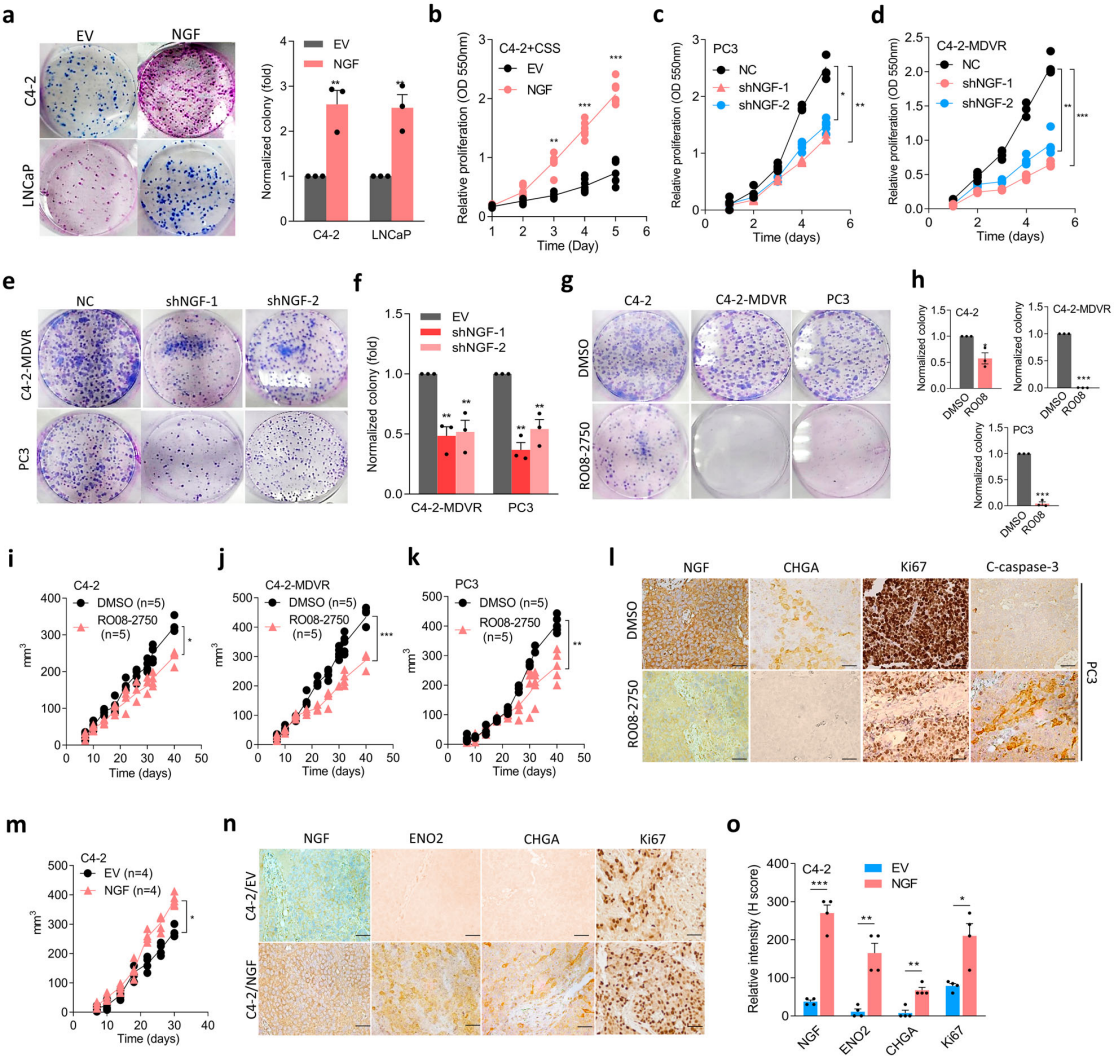
The NGF promotes therapeutic resistance and neuroendocrine differentiation of prostate cancer cells. **a** Images and quantification of the colony formation of C4-2 and LNCaP cells stably overexpressing an empty vector (EV) or NGF cDNA vector and cells treated with charcoal stripped serum (CSS)-containing medium for 1 week. * vs. the EV. **b** Proliferation assay of C4-2 cells treated with CSS-containing medium for 5 days following stable EV or NGF cDNA vector overexpression. * vs. the EV. **c**, **d** Proliferation assays of PC3 and C4-2-MDVR cells stably transfected with non-target control (NC) or NGF shRNA vectors. **e**, **f** Images (**e**) and quantification (**f**) of colony formation of C4-2-MDVR and PC3 cells stably expressing NC or NGF shRNA vectors. * vs. the NC. **g**, **h** Images (**g**) and quantification (**h**) of colony formation of C4-2, C4-2-MDVR, and PC3 cells treated with 10 μM RO08-2750 for 6 days. * vs. DMSO. Data from the colony formation and proliferation assays are presented as the mean ± SEM of three independent experiments; *n* = 8 per group. * *p* < 0.05, ** *p* < 0.01, *** *p* < 0.001; by a two-way ANOVA. **i**–**k** Tumor growth analysis of C4-2, C4-2-MDVR, and PC3 cells subcutaneously inoculated into male nude mice followed by treatment with 2.5 mg/kg RO08-2750 for 40 days. Tumor sizes were monitored twice a week (DMSO, *n* = 5 mice; RO08-2570, *n* = 5 mice). *p < 0.05, **p < 0.01, ***p < 0.001; by a two-way ANOVA. **l** IHC staining of subcutaneous tumors with antibodies specific for the NGF, CHGA, Ki67, and cleaved (C)-caspase-3 in tumor-bearing mice from (**k**). Scale bars, 100 µm. Statistical analysis was performed by a two-tailed Student’s *t*-test. *p < 0.05, **p < 0.01; by a two-way ANOVA. **m** Tumor growth analysis of C4-2 cells stably expressing an EV or NGF cDNA vector subcutaneously inoculated into male nude mice for 30 days. Tumor sizes were monitored twice a week (EV, *n* = 4 mice; NGF, *n* = 4 mice). ** *p* < 0.01; by a two-way ANOVA. **n**, **o** IHC staining (**n**) and relative intensities (**o**) of subcutaneous tumors with antibodies specific for the NGF, ENO2, CHGA, and Ki67 in tumor-bearing mice from (**m**). Scale bars, 100 µm. Statistical analysis was performed by a two-tailed Student’s *t*-test. *p < 0.05, **p < 0.01; by a two-way ANOVA.

To determine whether the NGF plays a role in tumor growth, we next analyzed cell proliferation effects in LNCaP and C4-2 cells with NGF overexpression. Overexpression of the NGF significantly increased colony formation compared to cells that carried the EV (Fig. 4a). We then cultured LNCaP and C4-2 cells harboring the NGF cDNA vector in CSS-containing medium or combined with MDV3100 treatment. Notably, overexpression of the NGF in cells promoted cell proliferation regardless of CSS-containing medium treatment (Fig. 4b; Supplementary Fig. 4a), whereas NGF-knockdown in AR-negative PC3 and ADT-resistance C4-2-MDVR cells reduced cell proliferation (Fig. 4c, d) and colony formation (Fig. 4e, f) compared to cells carrying the control vector. NGF expression in cells stably transfected with NGF cDNA or an shRNA vector was analyzed by Western blotting (Supplementary Fig. 4b, c). The role of the NGF was further examined in C4-2, C4-2-MDVR, and PC3 cells after treatment with the selective NGF inhibitor, RO08-2750^31,32^. Despite RO08-2750 being shown to reduce NGF expression (Supplementary Fig. 4d), we found that C4-2-MDVR and PC3 cells exhibited greater sensitivity to RO08-2750 compared to C4-2 cells or a normal prostate epithelial PZ-HPV-7 cells (Fig. 4g, h; Supplementary Fig. 4e). Results also showed that RO08-2750 was more effective on cells with NGF overexpression compared to cells transfected with an EV (Supplementary Fig. 4f, g), which suggests that RO08-2750 might potentially target cells with NGF overexpression. These results were further supported by in vivo experiments wherein mice were subcutaneously injected with C4-2, C4-2-MDVR, and PC3 cells and treated with RO08-2750. Mice which received RO08-2750 treatment exhibited a significant reduction in the tumor volume (Fig. 4i–k) and tumor weight (Supplementary Fig. 4h). We found that mice subcutaneously injected with tumors from C4-2-MDVR or PC3 cells exhibited an obvious effect of RO08-2750 compared to mice injected with C4-2 cells. Tumor formation was confirmed via IHC in C4-2-, PC3-, or C4-2-MDVR-injected mice, and we found that RO08-2750-treated groups showed significant decreases in NGF, CHGA, and proliferation (Ki67) marker levels and an increase in apoptotic marker (cleaved (C)-caspase-3) levels in PC3- or C4-2-MDVR-injected mice (Fig. 4l; Supplementary Fig. 4i–m). In contrast, when mice were injected subcutaneously with C4-2 cells stably expressing NGF cDNA, we found that the tumor size of mice increased (Fig. 4m). We harvested tissues from subcutaneous tumors and checked for neuroendocrine and proliferation markers, and results showed that tumors with NGF overexpression had increased neuroendocrine and proliferation marker expressions (Fig. 4n, o). Nevertheless, we found no significant metastatic tumors in these two groups. These data suggest that the NGF may act as a tumor promoter that drives ADT resistance and neuroendocrine differentiation of prostate cancer cells.

### Activated ZBTB46-NGF signaling is associated with CHRM4 stimulation

**Fig. 5:**
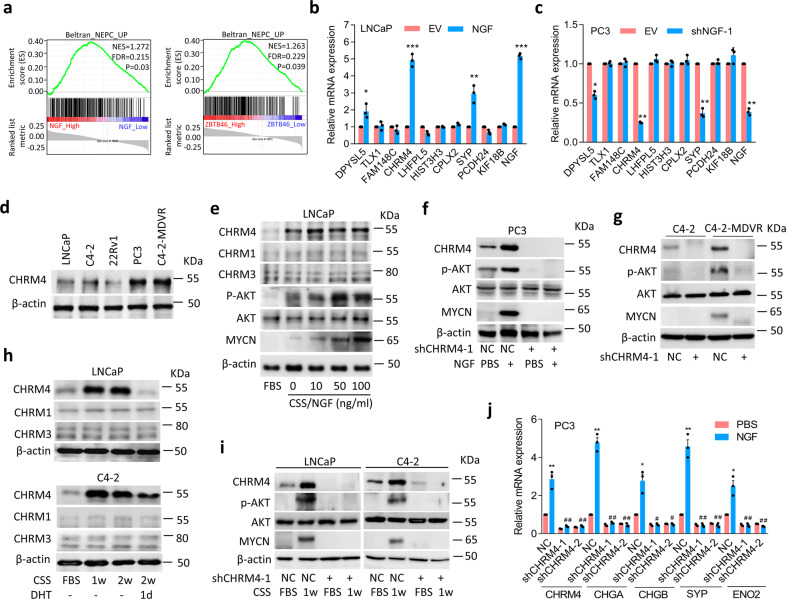
CHRM4 activates AKT and MYCN signaling and promotes neuroendocrine differentiation of prostate cancer. **a** GSEAs of TCGA prostate cancer dataset showed that higher expression levels of both the NGF and ZBTB46 in prostate cancer tissues were positively associated with a NEPC-responsive gene signature^25^. NES normalized enrichment score, FDR false discovery rate. **b**, **c** Relative mRNA levels of the top 10 candidate genes from Supplementary Table 6 in LNCaP cells with an empty vector (EV) or NGF cDNA vector overexpression (**b**) or in PC3 cells with non-target control (NC) or NGF shRNA vector expressions (**c**). **d** Western blotting of CHRM4 in various prostate cancer cell lines. **e** Western blotting of CHRM4, CHRM1, CHRM3, p-AKT, AKT, and MYCN in LNCaP cells treated with various concentrations of the NGF protein in charcoal-stripped serum (CSS)-containing medium for 1 week. Fetal bovine serum (FBS)-containing medium without the NGF protein served as a control. **f** Western blotting of CHRM4, p-AKT, AKT, and MYCN in PC3 cells stably transfected with the NC or CHRM4 shRNA vector, and cells were treated with 100 ng/ml NGF protein in CSS-containing medium for 1 week. **g** CHRM4, p-AKT, AKT, and MYCN protein levels in C4-2 and C4-2-MDVR cells following stable CHRM4-knockdown. **h** CHRM4, CHRM1, and CHRM3 protein levels in LNCaP and C4-2 cells cultured in CSS-containing medium for 1 and 2 weeks, followed by treatment with 10 nM dihydrotestosterone (DHT) for 1 day. **i** Protein levels of CHRM4, p-AKT, AKT, and MYCN in LNCaP and C4-2 cells following stable CHRM4-knockdown and cultured in CSS-containing medium for 1 week. **j** Relative mRNA levels of CHRM4, CHGA, CHGB, SYP, and ENO2 in PC3 cells following stable expression of NC or CHRM4 shRNA vector and treated cells with 100 ng/ml NGF protein in CSS-containing medium for 1 week. * vs. Phosphate buffered saline (PBS); ^#^ vs. the NC. Data from the quantification of mRNA are presented as the mean ± SEM of three independent experiments; *n* = 3 per group. *p < 0.05, **p < 0.01, ***p < 0.001; by a two-way ANOVA.

To identify the regulatory mechanisms between ZBTB46-NGF signaling and NEPC differentiation, we examined ZBTB46 and NGF expressions in gene signatures of NEPC in TCGA prostate cancer dataset by a GSEA. Results showed that tissues expressing high NGF and ZBTB46 levels were positively associated with upregulated NEPC-responsive genes^25^ (Fig. 5a). Relationships among NGF, ZBTB46, and NEPC-responsive genes were analyzed by a GSEA and showed that *DPYSL5, TLX1, FAM148C*, *CHRM4*, *LHFPL5*, *HIST3H3*, *CPLX2*, *SYP*, *PCDH24*, and *KIF18B* were the top 10 upregulated genes involved in increased NGF and ZBTB46 in both assays (Supplementary Data 1). In order to confirm relationships between expressions of these genes and the NGF, we overexpressed or knocked-down the NGF in cells and analyzed changes in these genes by examining their mRNA levels. We found that CHRM4 mRNA levels were positively associated with SYP upregulation and significantly increased in NGF-overexpressing LNCaP and C4-2 cells (Fig. 5b; Supplementary Fig. 5a), whereas reduced CHRM4 and SYP mRNA levels were observed in NGF-knockdown PC3 and C4-2-MDVR cells (Fig. 5c; Supplementary Fig. 5b). Next, we analyzed CHRM4 expression in prostate cancer cell lines and observed increased CHRM4 protein levels in PC3 and C4-2-MDVR cells (Fig. 5d). Since the activated PI3K/AKT pathway was reported to cross-talk with stimulated muscarinic receptor signaling^33,34^, and activated AKT is associated with MYCN expression in contributing to NEPC transformation^35^, we hypothesized that stimulation of the NGF–CHRM4 axis might upregulate AKT-MYCN signaling in prostate cancer. We found that overexpression of the NGF protein upregulated protein levels of CHRM4, phosphorylated (p)-AKT, and MYCN in LNCaP cells (Fig. 5e), whereas reductions in CHRM4, p-AKT, and MYCN were observed in CHRM4-knockdown PC3 cells, regardless of exogenous NGF protein treatment (Fig. 5f). Importantly, MDV3100-resistant C4-2 cells showed increases in CHRM4, p-AKT, and MYCN levels; however, knockdown of CHRM4 decreased p-AKT and MYCN expressions (Fig. 5g). These results suggest a role for NGF in NEPC progression, which is mechanistically linked to activation of CHRM4 and stimulation of the AKT–MYCN pathway. Results also showed that LNCaP and C4-2 cells with NGF protein treatment (Fig. 5e) or NGF cDNA overexpression (Supplementary Fig. 5c) exhibited significantly increased protein levels of CHRM4 but not CHRM1 or CHRM3. In order to assess whether CHRM4 upregulation is mediated by ADT, we validated CHRM1, CHRM3, and CHRM4 expressions in AR-positive LNCaP and C4-2 cells, relative to the AR signaling response. We found that CHRM1 and CHRM3 were not responsive to ADT, but CHRM4 significantly increased in cells subjected to CSS-containing medium treatment and was significantly reduced after DHT treatment (Fig. 5h). These data suggest that the role of CHRM4 in prostate cancer may differ from those of CHRM1 and CHRM3. Acetylcholine is a natural ligand for cholinergic receptors^36^. We found that acetylcholine increased expressions of CHRM1, CHRM3, and CHRM4, but no significant changes in neuroendocrine markers in LNCaP and C4-2 cells were observed (Supplementary Fig. 5d, e). Since we showed that upregulated NGF cannot increase expression of CHRM1 and CHRM3 (Fig. 5e; Supplementary Fig. 5c), this result suggests that NGF–CHRM4 might be a unique signaling pathway involved in neuroendocrine differentiation of prostate cancer that differs from canonical acetylcholine–CHRM pathways.

To study downstream signaling of CHRM4 after ADT, experiments were performed in LNCaP and C4-2 cells transfected with CHRM4 shRNA and further treated with CSS-containing medium. Western blotting revealed that CHRM4 accumulation was associated with increased p-AKT and MYCN levels following ADT; this effect was abolished in CHRM4-knockdown cells regardless of ADT (Fig. 5i). Moreover, the GSEA validated that tissues expressing high levels of both NGF and CHRM4 were positively associated with gene signatures responsive to activated AKT^37^ (Supplementary Fig. 5f) and MYCN-targeted^38^ (Supplementary Fig. 5g) signaling pathways. These results are consistent with the notion that the NGF upregulates CHRM4, through which it activates AKT-MYCN signaling after ADT. Moreover, ectopic CHRM4 cDNA expression upregulated neuroendocrine markers in LNCaP and C4-2 cells (Supplementary Fig. 5h, i), whereas knockdown of CHRM4 reduced neuroendocrine marker expressions in PC3 cells (Supplementary Fig. 5j). Importantly, CHRM4 and neuroendocrine markers accumulated following NGF protein treatment in PC3 cells; however, CHRM4-knockdown cells exhibited decreased neuroendocrine marker expressions even in cells with NGF protein treatment (Fig. 5j). We also found that MDV3100-resistant C4-2 cells exhibited upregulated levels of CHRM4 and neuroendocrine markers; however, reductions in neuroendocrine markers were observed in cells with CHRM4-knockdown (Supplementary Fig. 5k). These findings indicate that CHRM4 upregulation is associated with NEPC differentiation in an NGF-dependent manner.

### NGF is associated with CHRM4 and was shown to be upregulated in high-grade and SCNC samples

**Fig. 6:**
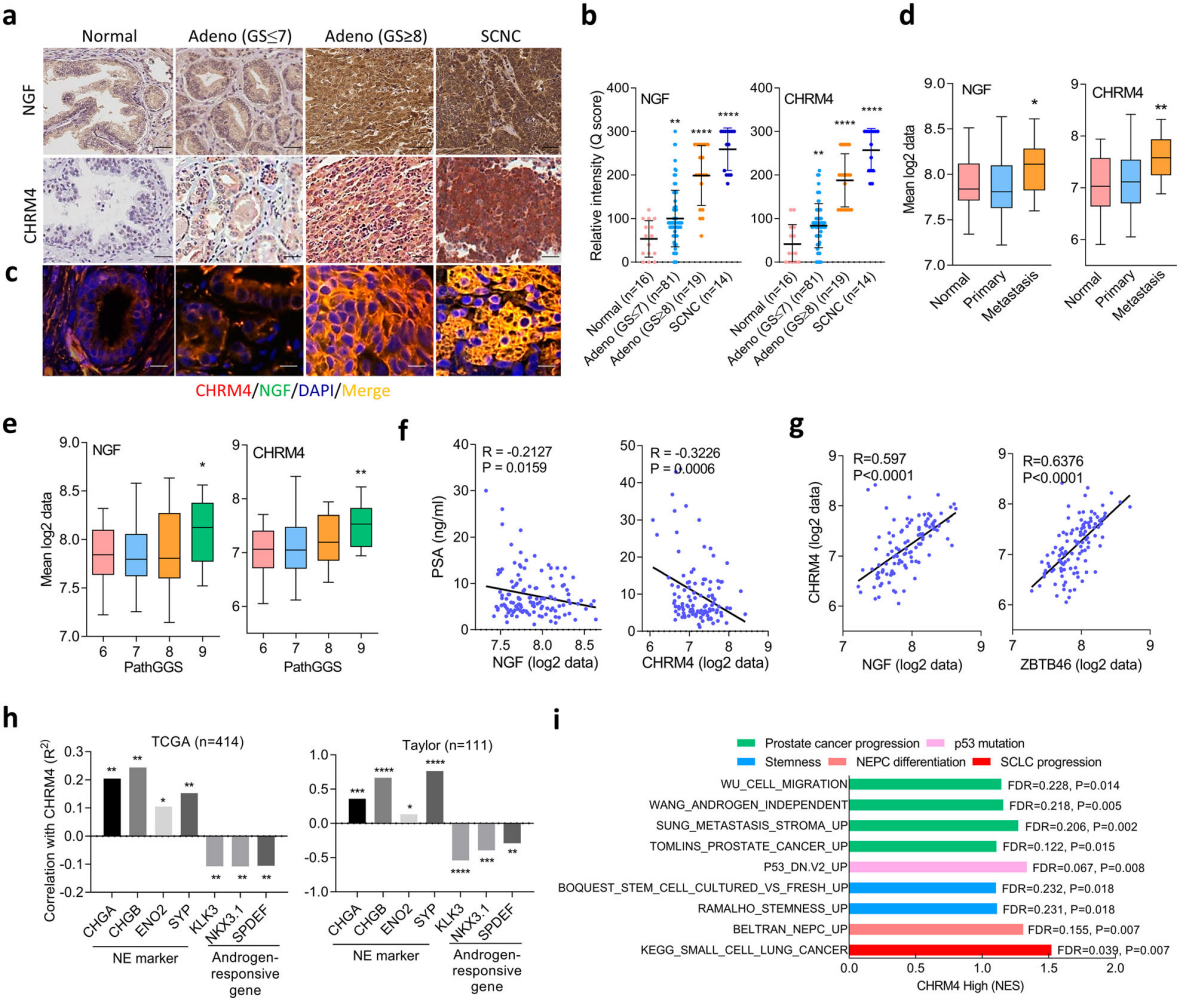
Positive associations among the NGF, CHRM4, and neuroendocrine markers in clinical samples. **a, b** IHC staining (**a**) and relative intensities (**b**) of the NGF and CHRM4 of a prostate cancer TMA consisting of normal tissues (*n* = 16), adenocarcinomas with a Gleason score of ≤7 (*n* = 81), adenocarcinomas with a Gleason score of ≥8 (*n* = 19), and SCNCs (*n* = 14) from Duke University School of Medicine. Scale bars, 100 μm. Data are presented as the mean ± SEM. * vs. normal tissues. *p < 0.05, ****p < 0.0001; by a two-way ANOVA. **c** Immunofluorescence staining of the tissue microarray (TMA) with antibodies for the CHRM4 (red) and NGF (green). Nuclei were visualized with DAPI staining (blue). Scale bars represent 20 µm. **d** Mean mRNA expression levels of the NGF and CHRM4 in human normal prostate (*n* = 28), primary (*n* = 98), and metastatic (*n* = 13) prostate cancer samples from the Taylor prostate cancer dataset^30^. * vs. normal tissues. *p < 0.05, **p < 0.01; by a two-way ANOVA. **e** Mean mRNA expressions of the NGF and CHRM4 in patient samples in the Taylor prostate cancer dataset^30^ by Gleason scores. * vs. Gleason score of 6. Significance was determined by a one-way ANOVA. **f**, **g** Correlation analysis of NGF and CHRM4 mRNA levels with PSA levels (**f**) and correlation analysis of NGF and ZBTB46 mRNA levels with CHRM4 mRNA levels (**g**) in prostate tissue samples from the Taylor prostate cancer dataset^30^. *R* correlation coefficient, *P* *p* (two-tailed) value. Data were tested by correlation XY analyses in GraphPad Prism. **h** Correlation analysis of CHRM4 mRNA levels with neuroendocrine marker and androgen-responsive gene mRNA levels in clinical tissue samples from TCGA and the Taylor prostate cancer datasets. *p < 0.05, **p < 0.01; by correlation XY analyses in GraphPad Prism. **i** GSEAs of TCGA prostate cancer dataset showing that higher CHRM4 expression of prostate tissues was significantly associated with prostate cancer progression^39–42^, p53 mutation^43^, stemness^44,45^, NEPC differentiation^25^, and SCLC progression (KEGG) gene signatures. NES normalized enrichment score, FDR false discovery rate.

To further study correlations between NGF and CHRM4 in human prostate tissues, we analyzed 16 normal prostatic epithelial samples, 81 primary low-grade prostate adenocarcinomas, 19 primary high-grade prostate adenocarcinomas, and 14 SCNCs from a prostate tissue microarray (TMA) collected from the Department of Pathology at Duke University School of Medicine (Durham, NC, USA). IHC analyses revealed that cytoplasmic NGF was associated with increased cytoplasmic CHRM4 and was highly expressed in high-grade tumors and SCNC samples (Fig. 6a, b). Immunofluorescence staining of CHRM4 and the NGF from the TMA was performed to validate the co-localization of CHRM4 and the NGF. Results showed that CHRM4 was co-localized with the NGF, and increased intensity was found in high-grade and SCNC samples, supporting the association between CHRM4 and the NGF in human tissue samples (Fig. 6c; Supplementary Fig. 6). Moreover, patients whose prostate tumors showed high NGF and CHRM4 mRNA expression levels also exhibited high metastatic potential (Fig. 6d) and a high pathological grade based on the Gleason score (PathGGS) (Fig. 6e), as validated in the Taylor prostate cancer datasets^30^. Furthermore, tumors exhibited upregulation of NGF and CHRM4 mRNA expressions were inversely associated with patients with low prostate-specific antigen (PSA) levels in the Taylor clinical prostate cancer dataset^30^ (Fig. 6f). The mean expression correlation was analyzed in prostate cancer datasets, which showed that CHRM4 was positively correlated with NGF, ZBTB46 (Fig. 6g), and neuroendocrine marker expressions and inversely correlated with androgen-responsive gene expressions (Fig. 6h). The GSEA also validated that tissues expressing high levels of CHRM4 were more likely to be positively associated with gene signatures involved in prostate cancer progression^39–42^, p53 mutation^43^, stemness^44,45^, NEPC differentiation^25^, and SCLC progression (KEGG) (Fig. 6i). These results support that activation of NGF–CHRM4 signaling is connected to malignant progression and neuroendocrine differentiation of prostate cancer.

### NGF physically interacts with CHRM4 after ADT

**Fig. 7:**
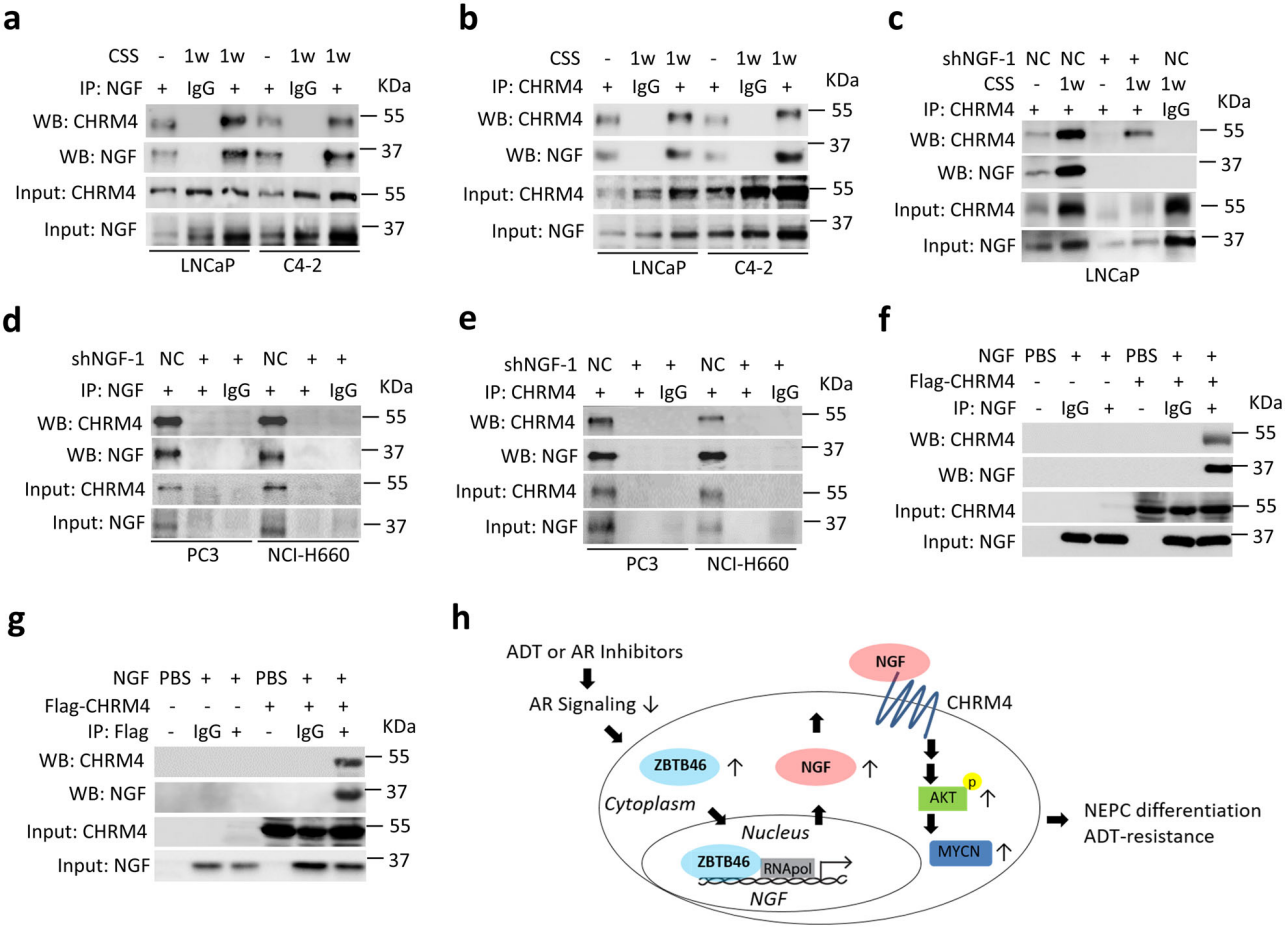
The NGF interacts with CHRM4. **a** Immunoprecipitation (IP) of the NGF and Western blotting of CHRM4 and the NGF in LNCaP and C4-2 cells cultured in charcoal stripped serum (CSS)-containing medium for 1 week. **b** IP of CHRM4 and Western blotting of CHRM4 and the NGF in LNCaP and C4-2 cells cultured in CSS-containing medium for 1 week. **c** IP of CHRM4 and Western blotting of CHRM4 and the NGF in LNCaP cells following stable NGF-knockdown and culturing in CSS-containing medium for 1 week. **d** IP of the NGF and Western blotting of CHRM4 and the NGF in PC3 and NCI-H660 cells stably expressing a non-target control (NC) or NGF shRNA vector. **e** IP of CHRM4 and Western blotting of CHRM4 and the NGF in PC3 and NCI-H660 cells stably expressing the NC or NGF shRNA vector. **f**, **g** IP of the NGF **f** or Flag **g** and Western blotting of CHRM4 and the NGF in a mixture of recombinant NGF protein and in vitro transcription/translation synthesized Flag-CHRM4 protein. **h** Proposed model of ADT or AR inhibitor-mediated therapeutic resistance and neuroendocrine differentiation of prostate cancer cells by stimulation of the ZBTB46 transcription factor, which upregulates the NGF. The upregulated NGF promotes ADT-resistance and NEPC development by integrating CHRM4 and activating the AKT–MYCN pathway.

To determine the possible interaction between NGF and CHRM4, AR-positive cells were subjected to ADT followed by an immunoprecipitation (IP)-Western blot analysis. A stable interaction was observed between the NGF and CHRM4 proteins in ADT-treated LNCaP and C4-2 cells by pulling down the NGF and immunoblotting with CHRM4 (Fig. 7a) and vice versa (Fig. 7b); however, a reduction in the interaction was observed in LNCaP and C4-2 cells with NGF-knockdown regardless of ADT treatment (Fig. 7c; Supplementary Fig. 7a). To confirm that this interaction also occurs in AR-negative cells, we validated the interaction between CHRM4 and the NGF in PC3 and NCI-H660 cells in response to NGF-knockdown. We found that this interaction was reduced in cells with NGF-knockdown by either pulling down the NGF or CHRM4 (Fig. 7d, e). Moreover, an established interaction was observed between a recombinant NGF protein and an in vitro transcription/translation-synthesized Flag-tag CHRM4 protein through pulling down the NGF or Flag and immunoblotting with CHRM4 or the NGF (Fig. 7f, g); however, this interaction was abolished when we pulled-down control immunoglobulin G (IgG). These observations confirm that the NGF physically interacts with CHRM4. Taken together, our findings support a model wherein ADT or AR inhibitor treatment stimulates ZBTB46 expression, which upregulates NGF-mediated CHRM4 stimulation; this plays a pivotal role in integrating AKT and MYCN signals to promote therapeutic resistance and neuroendocrine differentiation of prostate cancer (Fig. 7h).

## Discussion

CHRM4 is a muscarinic receptor, which is related to GPCRs^46,47^. Acetylcholine and conjugated secondary bile acids are principal ligands for endogenous muscarinic receptors^48^. Neurons produce acetylcholine, with well-characterized neurotransmitter properties and contributions to neuronal development via muscarinic receptors^49^. A recent study reported that nerve ending-derived acetylcholine induces CHRM1 activation in mesenchymal cells to promote prostate cancer invasion and metastasis^50^. The NGF, which is normally released from nerve endings, is a classical neurotransmitter in the central nervous system^6^. Overexpression of the NGF is significantly associated with a higher gastric cancer stage in an acetylcholine-CHRM3-dependent manner^51^. In prostate cancer, the NGF stimulates NTRK1 downstream of p38-MAPK activation to promote cell migration, invasion, and metastasis^11^. In our study, we established a link between NGF promotion of neuroendocrine differentiation via CHRM4 after the development of resistance to ADT in prostate cancer. Our study demonstrated that inhibition of AR signaling decreases activation of the NGF–CHRM4 axis, which is associated with neuroendocrine differentiation of prostate cancer, suggesting that current hormonal therapy designed to suppress AR functions may predispose prostate cancer to NEPC development. Our results suggest an interesting relationship between a classical neurotransmitter and a GPCR in neuroendocrine-differentiated prostate cancer cells. Activated NGF upregulates CHRM4 and links AKT signaling activation and MYCN stimulation to enhance NEPC reprogramming.

Several studies established that cancer progression requires neurogenesis^50,52,53^, which supports tumor development. Given the responses to prostate tumors, it is reasonable to assume that the NGF may also induce neuroendocrine differentiation of adenocarcinomas after ADT, which may affect tumor growth and progression in the tumor microenvironment^54,55^. Neurites were detected in 20% of breast tumors, and there is a possible association between NGF expression and the metastatic potential^56^. Indeed, it is clear that many types of tumors have the potential to secrete NGF, which induces peripheral nerve infiltration into the tumor microenvironment, thereby promoting tumor growth and metastasis^51,57^. A recent study demonstrated that the NGF is associated with genes involved in neuroendocrine differentiation from a gene expression profile of prostatic CXCR2^+^ neuroendocrine tumor cells, and it was also enriched in SCNC samples compared to CXCR2^−^ luminal prostate cancer cells and primary adenocarcinoma cases^58^. Whether neuroendocrine differentiation after ADT is related to upregulation of the NGF in prostate cancer is currently unclear. The loss of AR increased NGF expression in our study, suggesting that the AR may act as an upstream regulator that downregulates the NGF in the absence of ADT. AR inhibition by CSS or enzalutamide upregulates the NGF possibly because ADT inhibits the AR. We further knocked-down AR in AR-positive LNCaP cells using AR small interfering RNA, and found that knockdown of AR increased ZBTB46 and NGF expressions (Supplementary Fig. 7b). The AR may act upstream of both ZBTB46 and the NGF, and downregulates ZBTB46 and the NGF before ADT. Inhibition of AR signaling by androgen withdrawal or enzalutamide may abrogate the function of the AR, thereby upregulating both ZBTB46 and the NGF.

Canonical pathways triggered by interactions of the NGF with other receptors in neuroendocrine differentiation of prostate cancer are unknown. The association of the NGF with tumor progression has been widely studied in breast cancer^59^, melanomas^60^, pancreatic cancer^61^, and neuroblastomas^62,63^ through its two cognate receptors, NTRK1 and NGFR. In prostate cancer, the NGF has dual functions by interactions of its two cognate receptors, of which NTRK1 promotes aggressiveness^12^ and the NGFR may also reduce tumor growth^64^. In addition to NTRK1 and the NGFR, we identified a mechanism which is involved in ADT-mediated neuroendocrine differentiation in prostate cancer via CHRM4 upregulation. We demonstrated that the NGF physically interacts with CHRM4 and that the NGF mediates NEPC differentiation dependent on CHRM4. Importantly, we did not see increased or decreased levels of NGFR or NTRK1 in an ADT-mimicking condition or with additional androgen treatment (Supplementary Fig. 7c). This suggests that NTRK1 and the NGFR might not respond to AR signaling, and the roles of NGF–NGFR or NGF–NTRK1 signaling pathways might differ from that of NGF–CHRM4 signaling in prostate cancer. The specificity of the NGF–CHRM4 interrelationship can be used to develop specific drugs that target this interaction. It is conceivable that ADT-resistant or AR-negative cells could highly express CHRM4, and targeting CHRM4 may be a promising therapeutic strategy to treat NEPC. In addition, the NGF stimulates nerve infiltration into solid tumors and acts as a mediator of pain through activation of NTRK1 in the endings of sensory neurons^65^. Several studies demonstrated that blocking antibodies or pharmacological inhibitors against the NGF have a potent analgesic effect against pain^66–69^. Many prostate cancer patients exhibit bone pain if the prostate cancer spreads to the bones^67,70^. Therefore, targeting the NGF may have additional impacts of reducing malignant progression and bone pain in prostate cancer patients.

Our study results demonstrated a link between the NGF and NEPC differentiation through associations with CHRM4 via the ADT-upregulated ZBTB46 transcription factor. Thus, we discovered a mechanism of prostate cancer lineage plasticity that provides an effective prediction strategy utilizing gene expression-based biomarkers for NEPC development through the association of activated ZBTB46 and accumulated NGF via stimulation of the CHRM4–AKT–MYCN pathway. All recently approved drugs for NEPC and SCNC lack predictive biomarkers for selecting patient subgroups. Thus, our findings offer the potential to develop a prognostic information for current AR-directed therapeutic strategies with an antagonist of NGF–CHRM4 signaling.

## Methods

### Cells and reagents

Human normal prostate PZ-HPV-7 epithelial cells were obtained from the Bioresource Collection and Research Center (Hsinchu, Taiwan) and were cultured in keratinocyte serum-free medium (K-SFM) with 1% penicillin/streptomycin (Thermo Fisher, Waltham, MA, USA) and Normocin^®^ (Invitrogen, San Diego, CA, USA). The human prostate cancer LNCaP, C4-2, 22Rv1, and PC3 cell lines were obtained from American Type Culture Collection (ATCC; Manassas, VA, USA) and were cultured in RPMI 1640 medium supplemented with 10% FBS. The NEPC NCI-H660 cell line was purchased from ATCC and cultured in HITES medium composed of RPMI 1640 medium supplemented with insulin–transferrin–selenite (Sigma-Aldrich, St. Louis, MO, USA), 10 nM hydrocortisone (Sigma-Aldrich), 10 nM ß-estradiol (Sigma-Aldrich), 4 mM l-glutamine (Invitrogen), and 5% FBS. Culture medium was renewed thrice a week. As cell confluence reached 80%, cells were detached with 0.25% trypsin/EDTA (Invitrogen), collected in 15-ml canonical tubes (BD Biosciences, San Jose, CA, USA), and centrifuged at 1000 rpm for 5 min to eliminate trypsin. All experiments were completed in fewer than 20 passages to maintain uniformity. Cells were treated with 10 nM DHT (Sigma-Aldrich) and 100 ng/ml NGF (R&D Systems, Minneapolis, MN, USA) for 24 h in 10% CSS-containing medium. The AR antagonist, MDV3100 (Selleck Chemicals, Houston, TX, USA), and the NGF inhibitor, RO08-2750 (Selleck Chemicals), were used to treat cells at a concentration of 10 μM for 24 h in 10% FBS-containing medium. The MDV3100-resistant C4-2-MDVR cell line is a viable cell line generated by growing C4-2 cells under selection pressure of 20 μM MDV3100 for 6 months. Overexpression of ZBTB46, NGF, or CHRM4 was generated by establishment with a pCDH-CMV-MCS-EF1-Puro vector (System Biosciences, Palo Alto, CA, USA) encoding ZBTB46, NGF, or CHRM4 cDNA; an EV was used as a control. Knockdown of ZBTB46, NGF, or CHRM4 was generated by infection with a recombinant lentivirus encoding human ZBTB46, NGF, or CHRM4 shRNA (RNAi Core Lab, Taipei, Taiwan); a non-target control (NC) pLKO_TRC005-Puro vector was used as a control. The siRNAs (NC and siAR) were obtained from ON-TARGETplus SMARTpool siRNA (L-003400-00-0010, Thermo Scientific Dharmacon, Waltham, MA USA). Promoter reporters were constructed using the pGreenFire reporter (System Biosciences), and a Site-Directed Mutagenesis System kit (Invitrogen) was used for response element mutations. All primers used to generate these constructs are listed in Supplementary Table 1. All constructs were verified by a DNA sequence analysis.

### Real-time reverse-transcription (RT)-polymerase chain reaction (PCR)

An RNeasy Midi Kit (Qiagen, Redwood City, CA, USA) was used for total RNA isolation. For the RT-PCR, 1 µg of total RNA was used with a one-step real-time RT-PCR kit (Bio-Rad, Hercules, CA, USA). Reactions for all primer pairs were performed using a thermocycler at an initial temperature of 95 °C for 10 min, followed by 40 cycles of 95 °C for 15 s and 60 °C for 1 min. Normalization was performed by measuring human GAPDH expression, which was run in triplicate. All primers used for the PCR are listed in Supplementary Table 2.

### Western blot analysis

Cell lysates were extracted using radio immunoprecipitation assay buffer containing complete protease inhibitors (Roche, South San Francisco, CA, USA), phosphatase inhibitors (Roche), 25 mM β-glycerophosphate, 10 mM sodium fluoride, and 1 mM sodium vanadate. Twenty micrograms of cell lysate was then loaded onto each lane for separation via sodium dodecylsulfate gel electrophoresis, after which proteins were transferred to polyvinylidene difluoride or nitrocellulose membranes. Membranes were blocked with 5% bovine serum albumin in Tris-base buffer containing 0.1% Tween-20 and incubated overnight with primary antibodies at 4 °C. Secondary antibodies were then applied and incubated at room temperature for 1 h. All antibodies used are listed in Supplementary Table 3.

### RNA-Seq analysis

Transcriptome analyses of LNCaP/EV and LNCaP/NGF were conducted by BioTools (Xizhi, New Taipei, Taiwan), and the complete protocol was provided by Biotools. The complete process of the transcriptome analysis included three steps: RNA extraction and sequencing, data filtration and mapping, and an ontology analysis. Briefly, an mRNA and cDNA library was prepared by KAPA mRNA HyperPrep Kits (Roche) and sequenced using the NovaSeq^TM^ 6000 sequencing system (Illumina, San Diego, CA, USA) in 300 to 400-bp paired-end reads. In the data modulation step, the quality of raw reads was checked by FastQC and MultiQC, and then raw-paired end reads were trimmed with Trimommatic v0.38 (Leading: 3; Trailing: 3; Slidingwindow: 4:15; Minlen: 30) following by DIAMOND to detect contamination^71–73^. Clean reads were mapped to the reference genome (*Homo sapiens* GRCh38) by HISAT2 v2.1.0^74^. FeatureCount v1.6.0^75^ was used to map count readings to an individual gene. Read counts underwent relative log expression normalization by the DESeq2 v1.22.1 package in R^76^. Parameters and codes from each R package followed the default except for those mentioned above. Differentially expressed genes (DEGs) were determined using DESeq2 the threshold of significance of which was set to log (fold change) of >2 and *p*-adjusted of < 0.05 based on Benjamini and Hochberg’s approach^77^. A Gene Ontology analysis and KEGG pathway enrichment analysis of DEGs were carried out using clusterProfiler v3.10.1^78^. A GSEA was carried out with 1000 permutations to identify activated enriched pathways from MSigDB^79^.

### Dataset analyses

This study used TCGA prostate cancer dataset which consisted of level 3 normalized microarray gene expression data (UNC_AgilentG4502A_07) from TCGA and mRNA expression data for 414 primary prostate cancer samples from patients treated with a radical prostatectomy under National Cancer Institute, National Institutes of Health Review Board approval. The study used the Taylor prostate cancer dataset^30^, which was accessed from the Memorial-Sloan Kettering Cancer Center (MSKCC) Cancer Genomics data portal. We downloaded clinical and publicly available gene expression data on 28 normal prostate cancer samples, and 98 primary and 13 metastatic prostate cancer samples. The RNA-Seq dataset of paired prostate cancer samples pre-ADT and post-ADT was download from GEO (GSE48403). Expression data were log2-normalized. GSEA software was downloaded from the Broad Institute^80^, and gene sets of androgen-upregulated^27^^,28^, androgen-downregulated^29^, neuronal developmental-responsive (KEGG, Gene Ontology, and REACTOME), NEPC-responsive^25^, prostate cancer progression^39–42^, AKT-upregulated^37^, MYCN amplification-targeting^38^, p53 mutation^43^, stemness^44,45^, and SCLC progression (KEGG) gene signatures were used to determine correlations with ZBTB46, NGF, and CHRM4 levels. A normalized enrichment score (NES) and false discovery rate (FDR) were calculated using the GSEA program. For *z*-score analyses, gene sets were scored by summing expression *z*-scores per tumor within the cohort. Tumors were mean-stratified by NGF or CHRM4 expression, and the mean expression of each gene was determined in each group. Correlations among mRNA levels of ZBTB46, NGF, CHRM4, neuroendocrine markers, and androgen-responsive genes were obtained from TCGA and the Taylor^30^ prostate cancer datasets. Cutoff values used to identify “ZBTB46-high”, “NGF-high”, and “CHRM4-high” patients were predetermined by half the number of patients from both the GSEA and *z*-score analyses.

### ChIP assay

For the ChIP assay, we used an EZ magna ChIP A kit (Millipore, Billerica, MA, USA) according to a modified protocol^81^. For each sample, cells were treated with 10 μM MDV3100 or 10 nM DHT as indicated for 10 h and used at a concentration of 10^7^ cells in 10-cm dishes. Nuclear extract preparation, IP, and DNA-purification steps were performed according to the manufacturer’s protocol. Next, a quantitative (q)PCR was performed in triplicate using 1 µL of eluted chromatin. Enrichment is presented as a percentage of the total input. ZBTB46-binding and non-ZBTB46-binding sites located on the *NGF* regulatory sequence were upstream of human chromosomes 1:115289598 and 1:115291813 at GRCh38. A positive ZBTB46-binding site was identified from the human *SNAI1* promoter reporter^23^. ChIP antibodies and PCR primers are listed in Supplementary Table 4.

### Promoter reporter assay

Promoter reporter assays were performed in 12-well plates (5 × 10^4^ cells/well) with 1 µg of wild-type (WT) or mutant (M) promoter reporter transiently transfected into PC3 or NCI-H660 cells stably expressing ZBTB46 shRNA or into C4-2 or LNCaP cells stably expressing a ZBTB46 cDNA vector. MDV3100 treatment or ADT was performed in C4-2 or LNCaP cells in 10% FBS-containing or 10% CSS-containing medium for 24 h at 10 μM and 10 nM, respectively. Reporter activity was analyzed as relative median fluorescence intensity values of green fluorescent protein and measured using a fluorescence-activated cell sorter (BD Biosciences, San Jose, CA, USA) with FACS Diva software (BD Biosciences), normalized to the value of the vehicle.

### IHC staining

We collected 18 prostate cancer samples from the same patients before and after ADT from Taipei Medical University-Wan Fang Hospital (Taipei, Taiwan). TMA sections, including 16 normal prostatic epithelial samples, 81 primary low-grade prostate adenocarcinomas, 19 primary high-grade prostate adenocarcinomas, and 14 SCNCs, were provided by Duke University School of Medicine (Durham, NC, USA). Written informed consent was obtained from all patients, and the tissue samples were used in accordance with the *Declaration of Helsinki* and U.S. Common Rule, and their use was approved by the Taipei Medical University-Joint Institutional Review Board (approval no. N201901040) and the Duke University School of Medicine-Institutional Review Board (protocol ID, Pro00070193). IHC staining of samples for the NGF, ZBTB46, CHRM4, CHGA, ENO2, Ki67, and C-caspase-3 was performed using antibodies listed in Supplementary Table 5. Pathological diagnoses and intensities were reconfirmed by three pathologists (Wei-Yu Chen, Qingfu Zhang, and Phui-Ly Liew) and classified as 0 (negative), 1+ (weakly positive), 2+ (moderately positive), and 3+ (strongly positive). Intensity scoring values (range 0–300) were calculated using the following formula: (% cells with an intensity of 1+) +2 × (% cells with an intensity of 2+) +3 × (% cells with an intensity of 3+).

### Immunofluorescence staining

In total, 5 × 10^4^ cells/well were inoculated into Millicell EZ slide multi-chamber slides (Merck KGaA, Darmstadt, Germany) and cultured overnight. The next day, all treatments were performed on these cells. After completing treatment, cells were sequentially fixed with 4% paraformaldehyde/PBS for 10 min and permeabilized with 0.1% Triton-X100/PBS for 5 min. Fixed cells were blocked using 5% BSA/0.1% tween-20/PBS (PBST) for 1 h and then stained with NGF (1:300, Abcam, ab52918, Cambridge, UK), ENO2 (1:50, Santa Cruz, sc21738, Dallas, TX, USA), SYP (1:250, Invitrogen, MA5-14523), and CHRM4 (1:200, Invitrogen, PA5-77483) antibodies at 4 °C overnight. Samples stained with the first antibody were labeled with an Alexa Fluor 488-conjugated anti-rabbit antibody (1:500, Invitrogen, A27034) and an Alexa Fluor 568-conjugated anti-mouse antibody (1:1000, Invitrogen, A11031) for 1 h and mounted using DAPI-Fluoromount-G™ mounting media (Electron Microscopy Sciences, Hatfield, PA, USA). Cellular images were taken with a fluorescence microscope (Olympus, Tokyo, Japan) and then merged using ImageJ software^82^.

### Proliferation assay

C4-2 and LNCaP cells stably expressing an EV or NGF cDNA vector were cultured in 10% CSS-containing medium, or NGF-knockdown PC3 and NCI-H660 cells were cultured in 10% FBS-containing medium. Cells were seeded at a density of 2 × 10^3^ cells/well in 96-well plates and analyzed using a Cell Proliferation Assay Kit (Promega, Madison, WI, USA) according to the manufacturer’s instructions. The experiment was performed with multiple wells at each time point and then averaged. The absorbance was quantified at a wavelength of OD 550 nm using a plate reader.

### Colony-formation assay

Single-cell suspensions of C4-2 and LNCaP cells stably expressing the NGF cDNA vector were used for colony-formation assays. Cells were seeded at a density of 500 cells/well in six-well plates and incubated for 7 days at 37 °C in a humidified incubator. Analysis was performed in triplicate. Following incubation in a 0.5% crystal violet fixative solution for 15 min, colonies of more than 50 μm in diameter were counted and quantified for each replicate performed in triplicate.

### Tumorigenicity assays in mice

For the tumorigenicity assays, 6-week-old male nude mice (NLAC, Taipei, Taiwan) were subcutaneously injected with 2.5 × 10^6^ C4-2, C4-2-MDVR, or PC3 cells in 50% Matrigel^®^ (BD Biosciences). Animal work was performed following a protocol approved by the Taipei Medical University Animal Care and Use Committee (approval no.: LAC-2018-0439, Taipei, Taiwan). Mice injected with C4-2, C4-2-MDVR, or PC3 cells were treated with 2.5 mg/kg RO08-2750 or DMSO (control) for 40 days via an intraperitoneal injection twice a week. For NGF overexpression, 2 × 10^6^ cells/mice of C4-2/EV or C4-2/NGF cells were subcutaneously injected into the right flank of mice and incubated for 30 days. The tumor size was measured twice a week with calipers, and the tumor volume was calculated using the following formula: (4/3) × (*L*/2) (*W*/2)^2^, where *L* is the length and *W* is the width.

### Statistics and reproducibility

All statistical analyses were performed using GraphPad Prism analytical tools (GraphPad Software, San Diego, CA, USA), and results are presented as the mean ± standard error of the mean (SEM). Statistical differences between individual groups were determined by a two-tailed *t*-test or one-way analysis of variance followed by Bonferroni’s post-test for comparisons among three or more groups. The method for determining cutoff values was pre-decided by half the number of patients in the *z*-score analyses. *p* values of < 0.05 were considered statistically significant.

## Supplementary information

Supplementary Information

Description of Supplementary Files

Supplementary Data 1

Supplementary Data 2

## Data Availability

Raw images of the western blots are provided in Supplementary Fig. 8. Raw RNA sequencing data are available at the NCBI Gene Expression Omnibus (GEO) under accession GSE161070. The data supporting the results of the study can be found in the manuscript file or obtained from the corresponding author upon reasonable request.
