# Supplementary material for: Nerve growth factor interacts with CHRM4 and promotes neuroendocrine differentiation of prostate cancer and castration resistance
Source: Commun Biol. 2021 Jan 4;4:22. doi: 10.1038/s42003-020-01549-1 (PMC7782543; doi:10.1038/s42003-020-01549-1)
Supplement: Supplementary file 1 — Supplementary Information [file 42003_2020_1549_MOESM1_ESM.pdf]

Supplementary Information

Supplementary figure

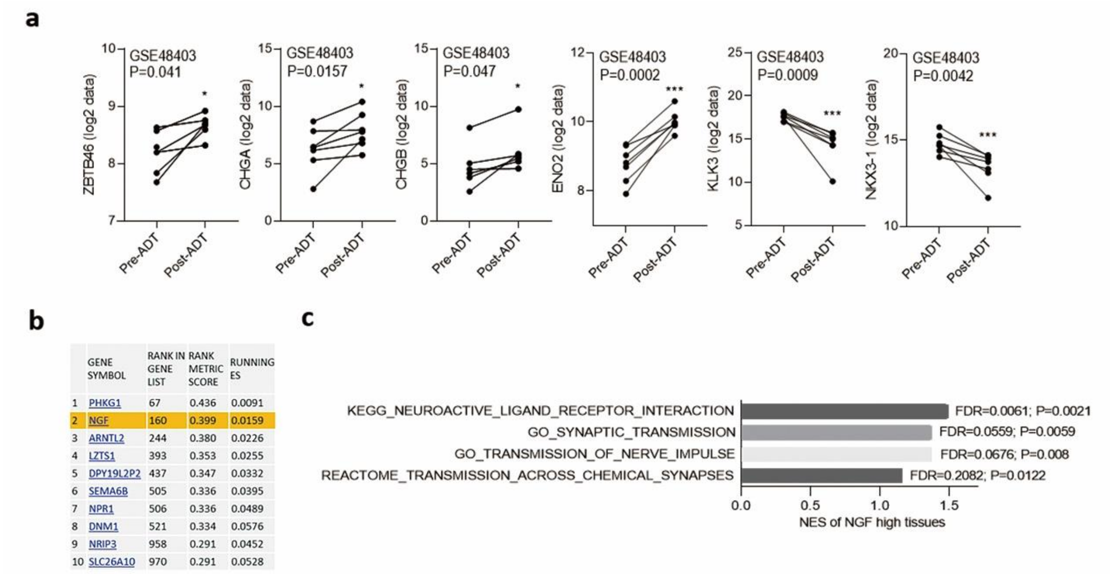

Supplementary fig. 1: ADT upregulates ZBTB46, which is involved in NE differentiation of prostate cancer. a Expressions of ZBTB46, NE markers (CHGA, CHGB, and ENO2), and androgen-responsive genes (KLK3 and NKX3-1) in paired prostate cancer samples pre- and post-ADT from the GSE48403 dataset. **b** *NGF* is a candidate gene from the list of genes that have upregulated NEPC-responsive gene signatures [1] and was positively associated with higher ZBTB46-expressing tissues by GSEA of TCGA prostate cancer dataset. **c** GSEAs of TCGA prostate cancer dataset showing enrichment of NGF expression among the gene sets, the expression levels of which were activated in association with neuronal developmental-responsive signatures (KEGG, GO, and REACTOME). NES, normalized enrichment score; FDR, false discovery rate.

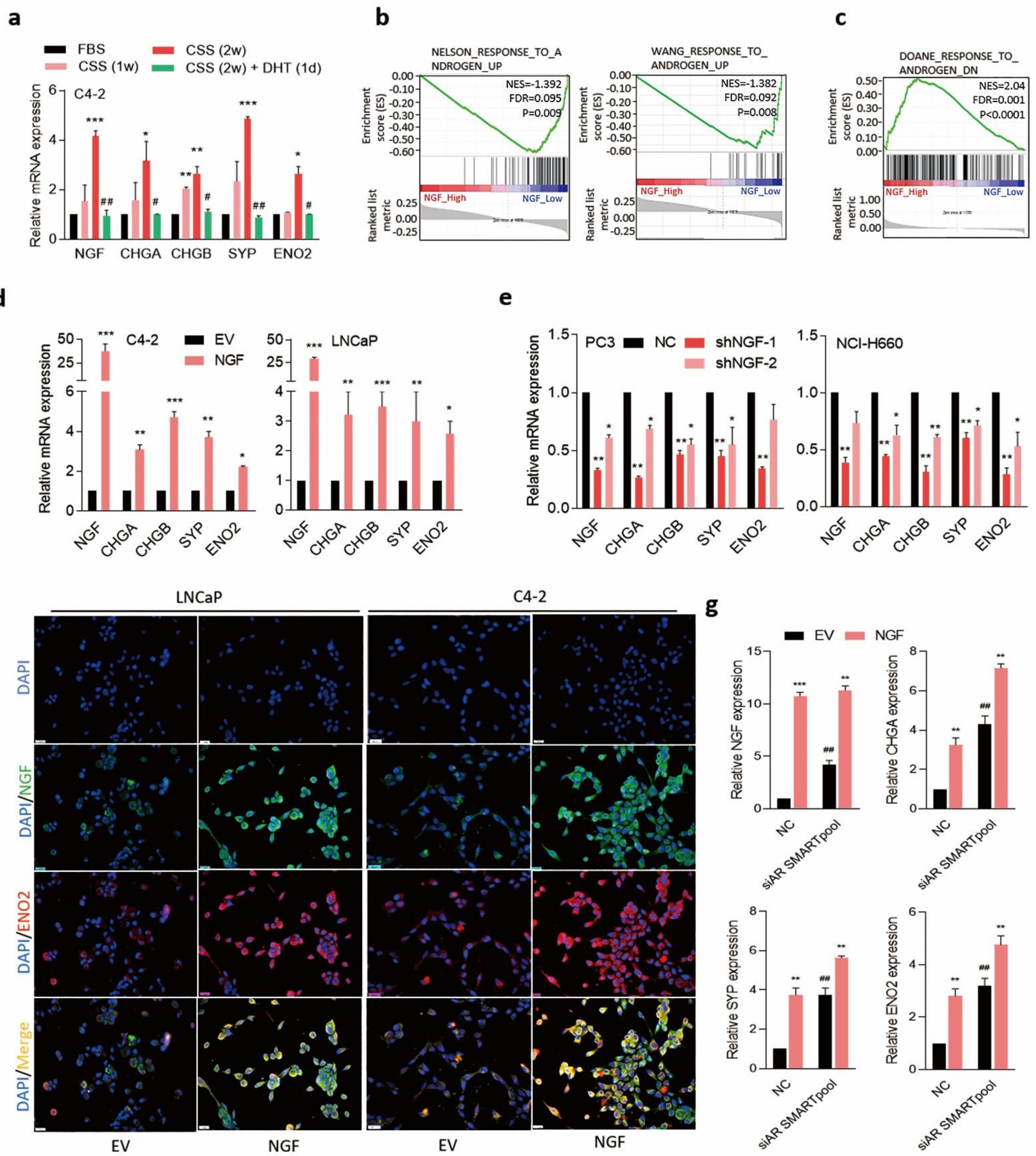
a
FBS
CSS (1w)
CSS (2w)
CSS (2w) + DHT (1d)
C4-2
Relative mRNA expression
NGF CHGA CHGB SYP ENO2
b
NELSON_RESPONSE_TO_ANDROGEN_UP
NES=-1.392 FDR=0.095 P=0.009
WANG_RESPONSE_TO_ANDROGEN_UP
NES=-1.382 FDR=0.092 P=0.008
Enrichment score (ES)
Ranked list metric
NGF_High
NGF_Low
c
DOANE_RESPONSE_TO_ANDROGEN_DN
NES=2.04 FDR=0.001 P<0.0001
d
C4-2
LNCaP
EV
NGF
Relative mRNA expression
e
PC3
NC
shNGF-1
shNGF-2
NCI-H660
f
LNCaP
C4-2
DAPI
DAPI/NGF
DAPI/ENO2
DAPI/Merge
EV
NGF
EV
NGF
g
EV
NGF
Relative NGF expression
Relative CHGA expression
Relative SYP expression
Relative ENO2 expression
NC
siAR SMARTpool

Supplementary fig. 2: ADT-induced NGF is associated with NE differentiation of prostate cancer. **a** Relative mRNA levels of the NGF, CHGA, CHGB, SYP, and ENO2 in C4-2 cells cultured in CSS-containing medium for 1 and 2 weeks and then treated with 10 nM DHT for 24 h. * vs. FBS; $^{\#}$ vs. CSS (2 weeks). **b** GSEA showing that lower NGF expression among patients in TCGA prostate cancer dataset was positively associated with two androgen-upregulated gene signatures [2,3]. NES, normalized enrichment score; FDR, false discovery rate. **c** GSEA of TCGA prostate cancer dataset showing that higher NGF expression was positively associated with an androgen-downregulated gene signature [4]. **d, e** Relative mRNA levels of the NGF, CHGA, CHGB, SYP, and ENO2 in C4-2 and LNCaP cells stably expressing an empty vector (EV) or NGF cDNA vector (**d**) or in PC3 and NCI-H660 cells with stable expression of non-target control (NC) or NGF shRNA vectors (**e**). **f** IF staining of LNCaP and C4-2 cells stably expressing an EV or NGF cDNA vector with antibodies for the NGF (green) and ENO2 (red). Nuclei were visualized with DAPI staining (blue). Scale bars represent 20 µm. **g** Relative mRNA levels of the NGF, CHGA, SYP, and ENO2 in LNCaP cells stably expressing an EV or an NGF cDNA expression vector following AR-knockdown by siAR SMARTpool. * vs. the EV; $^{\#}$ vs. the NC. Data from the quantification of mRNA are presented as the mean ± SEM; n=3 per group. * p<0.05, ** p<0.01, *** p<0.001; tested by a two-way ANOVA.

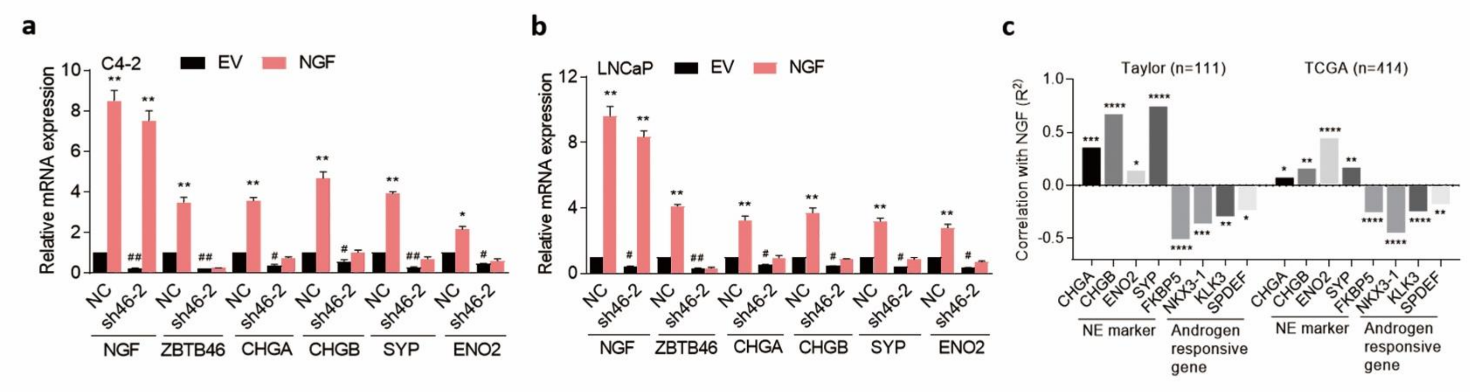

Supplementary fig. 3: The NGF upregulation is involved in NE differentiation of prostate cancer. a, b Relative mRNA levels of the NGF, ZBTB46, CHGA, CHGB, SYP, and ENO2 in C4-2 and LNCaP cells stably expressing an empty vector (EV) or NGF cDNA expression vector following non-targeted control (NC) or ZBTB46 shRNA vector expression. * vs. the EV; # vs. the NC. Data from the quantification of mRNA are presented as the mean ± SEM; n=3 per group. * p<0.05, ** p<0.01, *** p<0.001; tested by a two-way ANOVA. **c** Correlation analysis of *NGF* with NE markers and androgen-responsive gene mRNA levels in clinical tissue samples from the Taylor and TCGA prostate cancer datasets. Significance was determined by correlation XY analyses in GraphPad Prism. * p<0.05, ** p<0.01, *** p<0.001, **** p<0.0001.

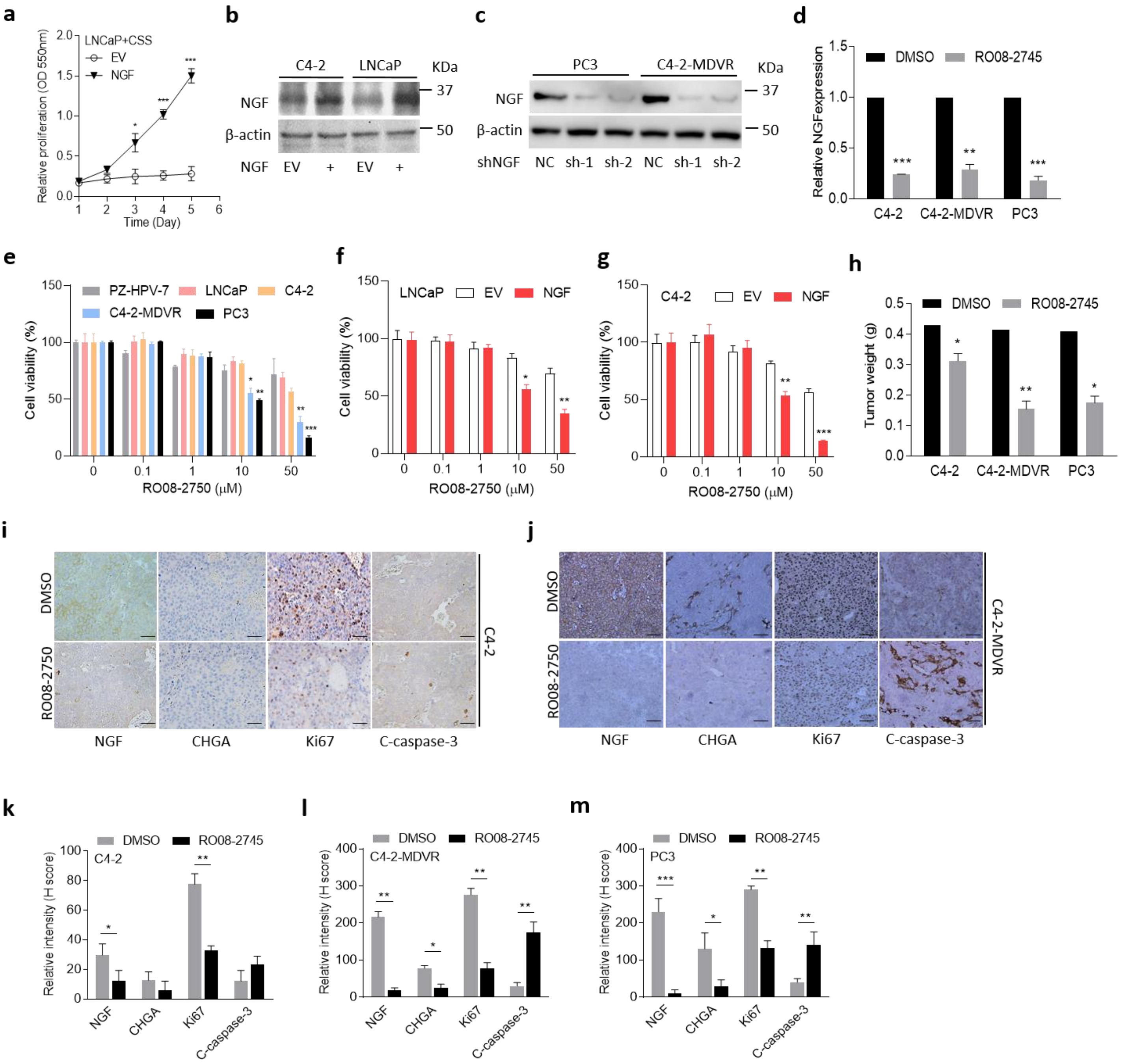

a
LNCaP+CSS
EV
NGF
Relative proliferation (OD 550nm)
Time (Day)
b
C4-2
LNCaP
KDa
NGF
β-actin
EV
+
37
50
c
PC3
C4-2-MDVR
shNGF
NC
sh-1
sh-2
d
DMSO
RO08-2745
Relative NGFexpression
C4-2
C4-2-MDVR
PC3
e
PZ-HPV-7
LNCaP
C4-2
C4-2-MDVR
PC3
Cell viability (%)
RO08-2750 (μM)
f
g
h
Tumor weight (g)
i
DMSO
RO08-2750
NGF
CHGA
Ki67
C-caspase-3
j
k
Relative intensity (H score)
l
m

Supplementary fig. 4: The NGF promotes cell proliferation and NE differentiation of prostate cancer. a Proliferation assay of LNCaP cells treated with CSS-containing medium for 5 days followed by stable expression of an empty vector (EV) or NGF cDNA vector. * vs. the EV. Data are presented as the mean ± SEM; $n = 8$ per group. * $p<0.05$, *** $p<0.001$; by a two-way ANOVA. **b, c** Western blotting of the NGF in C4-2 and LNCaP cells stably transfected with an EV or NGF cDNA vector (**b**) or in PC3 and C4-2-MDVR cells with a non-target control (NC) or NGF shRNA stable expression (**c**). **d** Relative NGF mRNA expression in C4-2, C4-2-MDVR, and PC3 cells treated with 10 μM RO08-2750 for 6 days. * vs. DMSO. **e** Proliferation assays of PZ-HPV-7, LNCaP, C4-2, C4-2-MDVR, and PC3 cells treated with RO08-2750 at the indicated concentrations for 24 h ($n = 8$). * vs. 0 μM RO08-2750. **f, g** Proliferation assays of LNCaP and C4-2 cells with an EV or NGF cDNA stable expression and treated with RO08-2750 at the indicated concentrations for 24 h ($n = 8$). * vs. 0 μM RO08-2750. **h** Weights of subcutaneously injected tumors from C4-2, C4-2-MDVR, and PC3 cells in mice at the end of the experiment from Fig. 4i-k. * vs. DMSO. **i-m** IHC staining (**i** and **j**) and relative intensities (**k-m**) of subcutaneous tumors with antibodies specific for the NGF, CHGA, Ki67, and cleaved (C)-caspase-3 in tumor-bearing mice from Fig. 4i-k. Scale bars, 100 µm. * vs. DMSO. Statistical analysis was performed by a two-tailed Student's *t*-test. * $p<0.05$, ** $p<0.01$; by a two-way ANOVA.

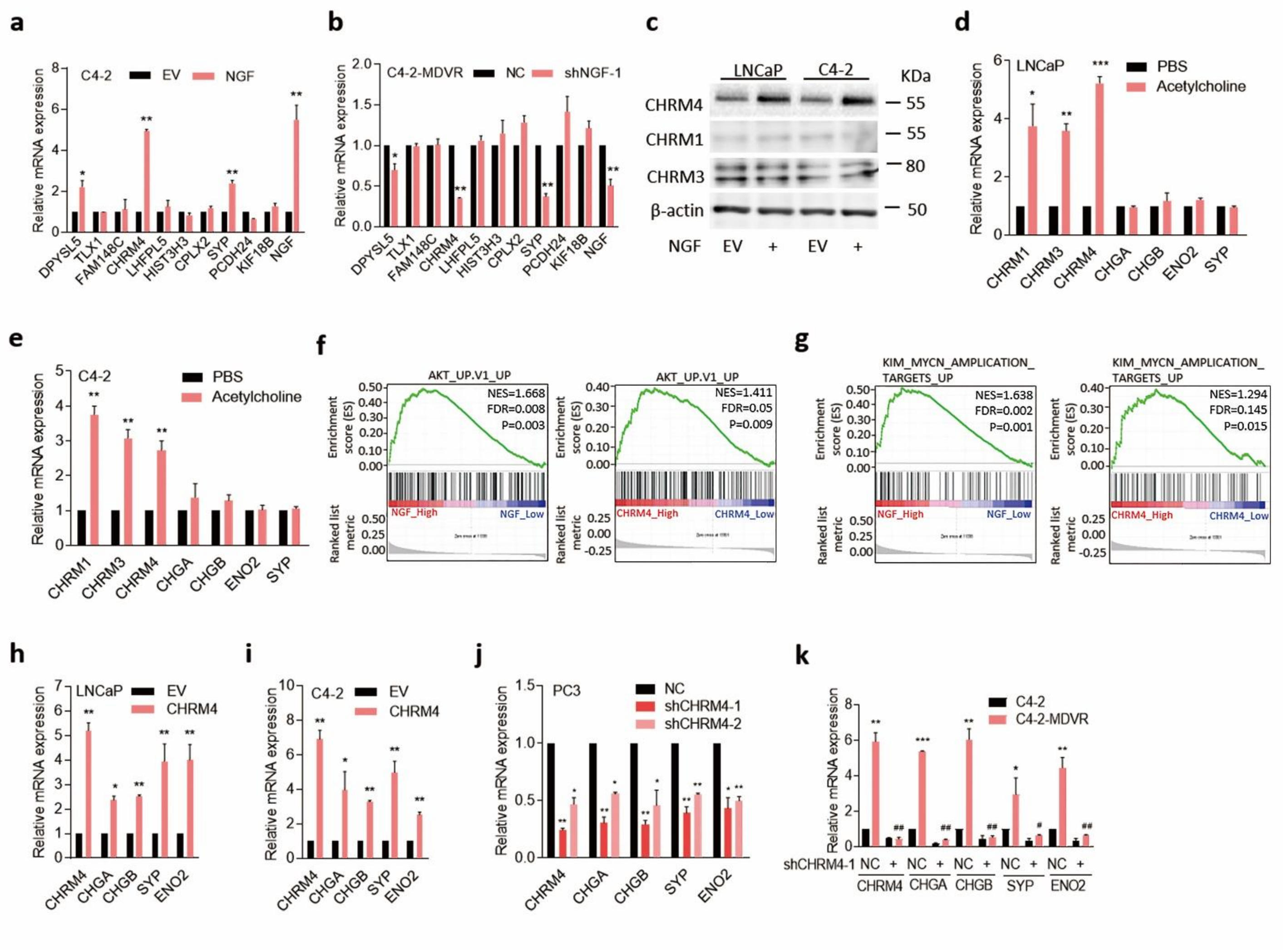
a
C4-2
EV
NGF
Relative mRNA expression
DPYSL5
TLX1
FAM148C
CHRM4
LHFPL5
HIST3H3
CPLX2
SYP
PCDH24
KIF18B
NGF
b
C4-2-MDVR
NC
shNGF-1
c
LNCaP
C4-2
KDa
CHRM4
CHRM1
CHRM3
β-actin
NGF
EV
+
d
LNCaP
PBS
Acetylcholine
CHRM1
CHRM3
CHGA
CHGB
ENO2
e
C4-2
f
AKT_UP.V1_UP
NES=1.668
FDR=0.008
P=0.003
NES=1.411
FDR=0.05
P=0.009
Enrichment score (ES)
Ranked list metric
NGF_High
NGF_Low
CHRM4_High
CHRM4_Low
g
KIM_MYCN_AMPLICATION_TARGETS_UP
NES=1.638
FDR=0.002
P=0.001
NES=1.294
FDR=0.145
P=0.015
h
LNCaP
i
j
PC3
shCHRM4-1
shCHRM4-2
k
C4-2-MDVR
shCHRM4-1

Supplementary fig. 5: Increase of CHRM4 is associated with upregulated-NGF and -ZBTB46 of prostate cancer. a, b Relative mRNA levels of top ten candidate genes from Supplementary table 6 in C4-2 cells with an empty vector (EV) or NGF cDNA vector overexpression (**a**) or in C4-2-MDVR cells with non-target control (NC) or NGF shRNA vectors expression (**b**). * vs. the EV or NC. **c** Western blotting of CHRM4, CHRM1, and CHRM3 in LNCaP and C4-2 cells stably transfected with an EV or NGF cDNA vector. **d, e** Relative mRNA levels of CHRM1, CHRM3, CHRM4, CHGA, CHGB, ENO2, and SYP in LNCaP and C4-2 cells treated with 10 μM acetylcholine in CSS-containing medium for 48 h. * vs. PBS. **f, g** GSEAs of the TCGA prostate cancer dataset showed that higher NGF and CHRM4 expressions by prostate cancer tissues were both positively associated with gene signatures involved in AKT signaling activation [5] (**f**) and MYCN amplification targets [6] (**g**). NES, normalized enrichment score; FDR, false discovery rate. **h, i** Relative mRNA levels of CHRM4, CHGA, CHGB, SYP, and ENO2 in LNCaP and C4-2 cells stably expressing an EV or CHRM4 cDNA vector. * vs. the EV. **j** CHRM4, CHGA, CHGB, SYP, and ENO2 mRNA levels in CHRM4-knockdown PC3 cells. * vs. the NC. **k** CHRM4, CHGA, CHGB, SYP, and ENO2 mRNA levels in C4-2 and C4-2-MDVR cells following stable CHRM4-knockdown. * C4-2 vs. C4-2-MDVR, $^{\#}$ vs. the NC. Data from the quantification of mRNA are presented as the mean ± SEM, $n = 3$ per group. * $p<0.05$, ** $p<0.01$, *** $p<0.001$; by a two-way ANOVA.

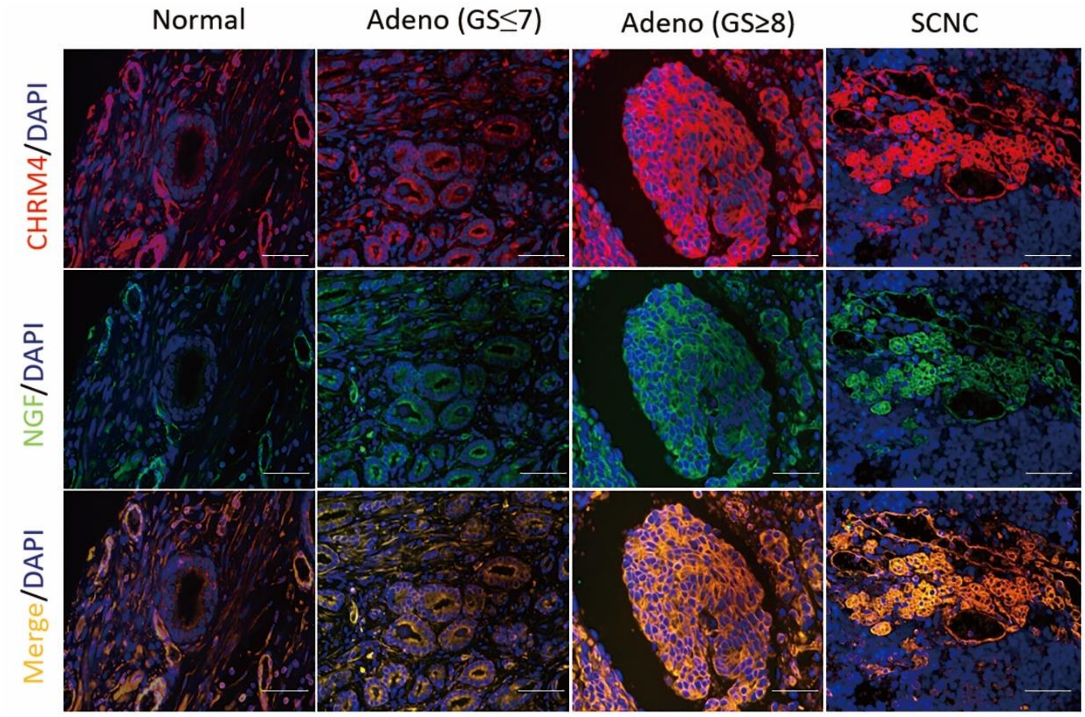

Supplementary fig. 6: CHRM4 co-localizes with the NGF. Images of the intensities of CHRM4 and NGF by IF staining in the TMA from Fig. 6a with antibodies for CHRM4 (red) and the NGF (green). Nuclei were visualized with DAPI staining (blue). Images showing the co-expression of CHRM4 and NGF in the same tumor cells. Scale bars, 100 μm.

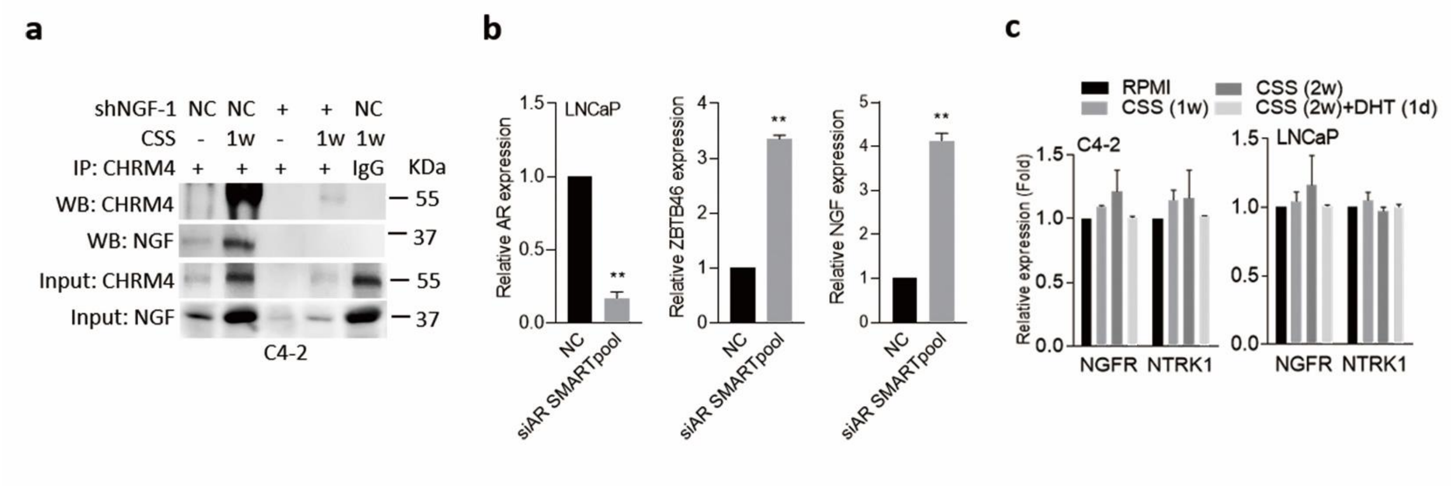

Supplementary fig. 7: ADT-induced-NGF interacts with CHRM4. a IP of CHRM4 and Western blotting of CHRM4 and NGF in C4-2 cells following stable NGF-knockdown and culture in CSS-containing medium for 1 week. **b** Relative mRNA levels of the AR, ZBTB46, and NGF in LNCaP cells following AR-knockdown by siAR SMARTpool. * vs. a non-target control (NC). **c** Relative mRNA levels of the NGFR and NTRK1 in C4-2 and LNCaP cells cultured with CSS-containing medium for 1 and 2 weeks and then treated with 10 nM DHT for 24 h. Data from the quantification of mRNA are presented as the mean ± SEM; *n*=3 per group. ** *p*<0.01; tested by a two-way ANOVA.

Supplementary fig. 8.

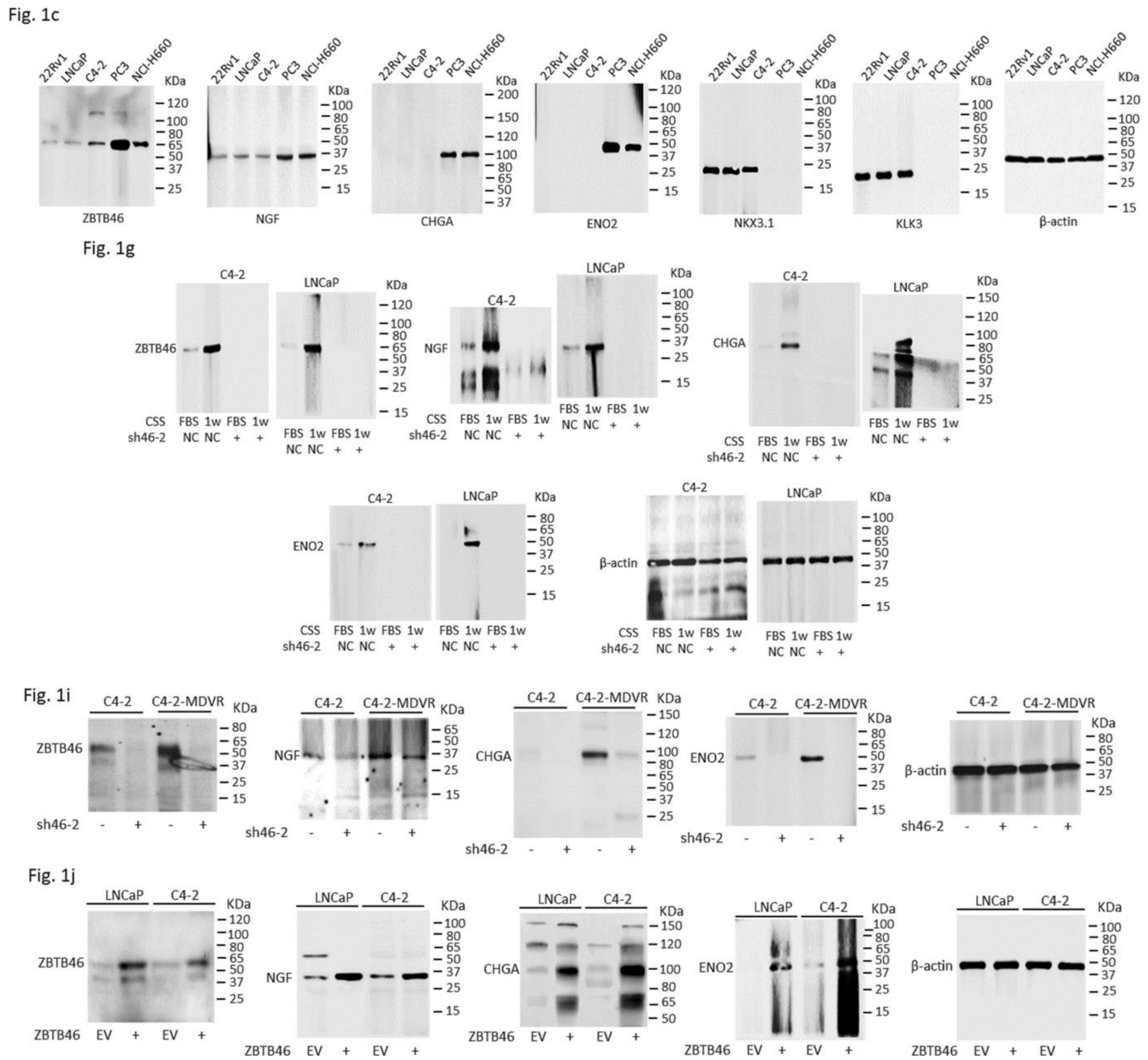

Fig. 3d

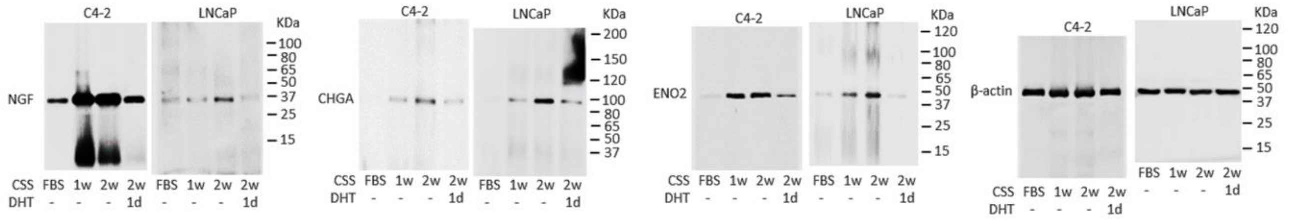

Fig. 3e

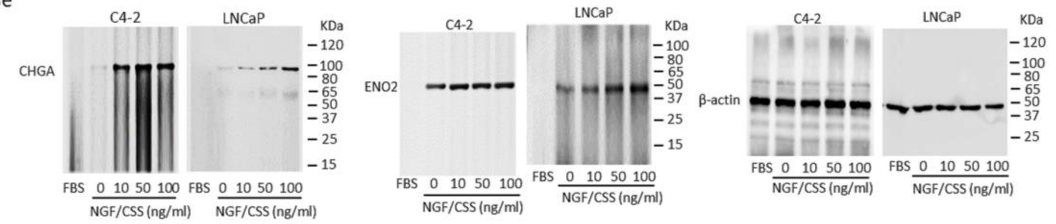

Fig. 3f

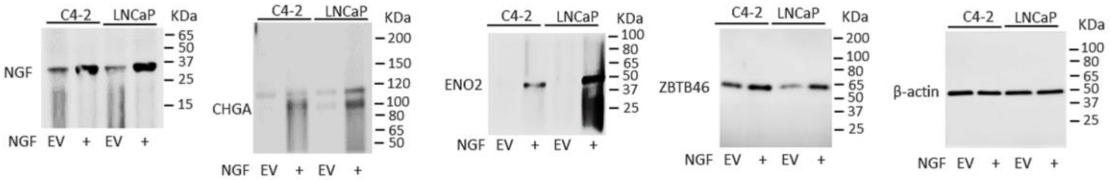

Fig. 3g

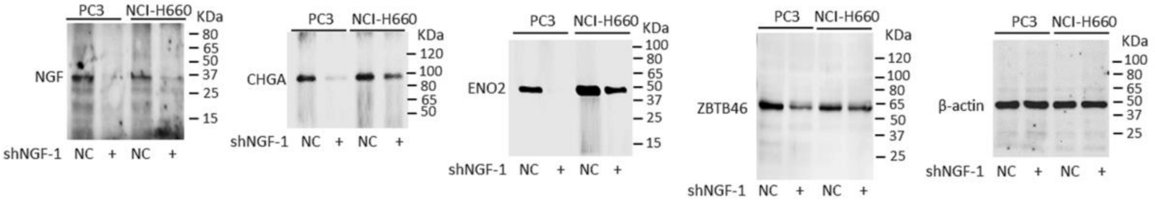

Fig. 3h

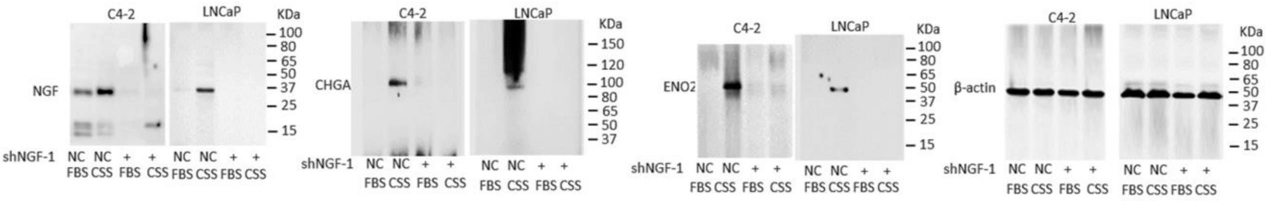

Fig. 5d

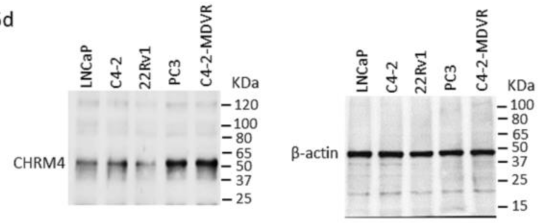

Fig. 5e

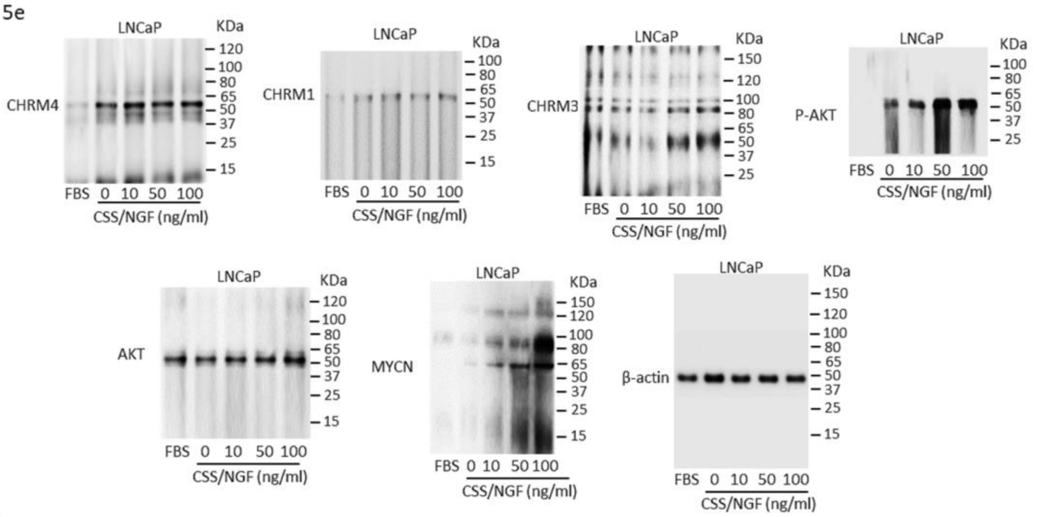

Fig. 5f

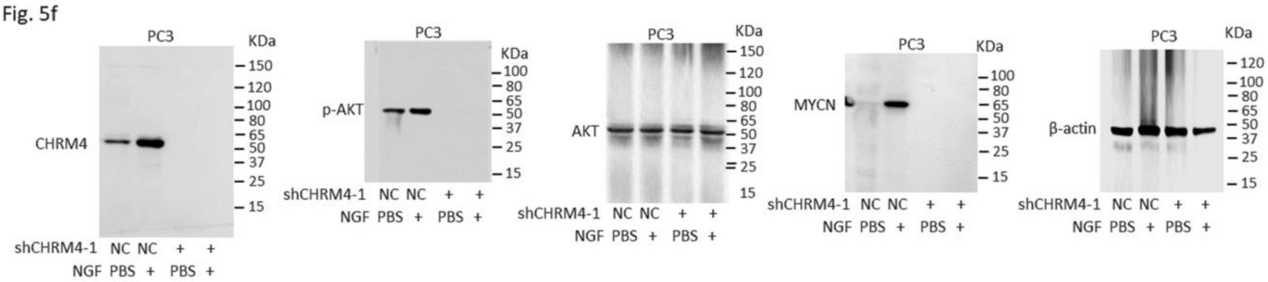

Fig. 5g

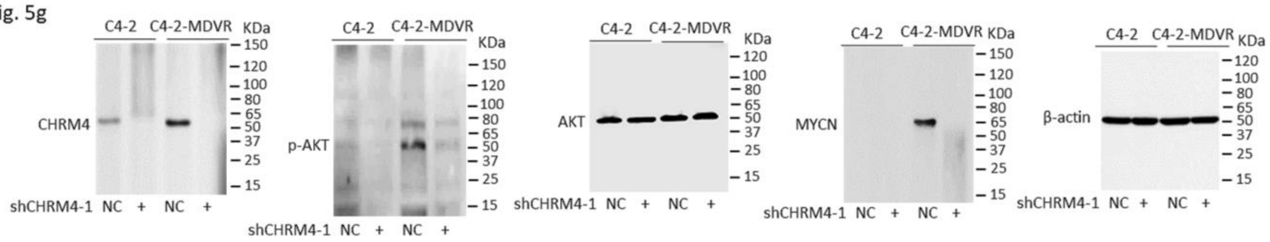

Fig. 5h

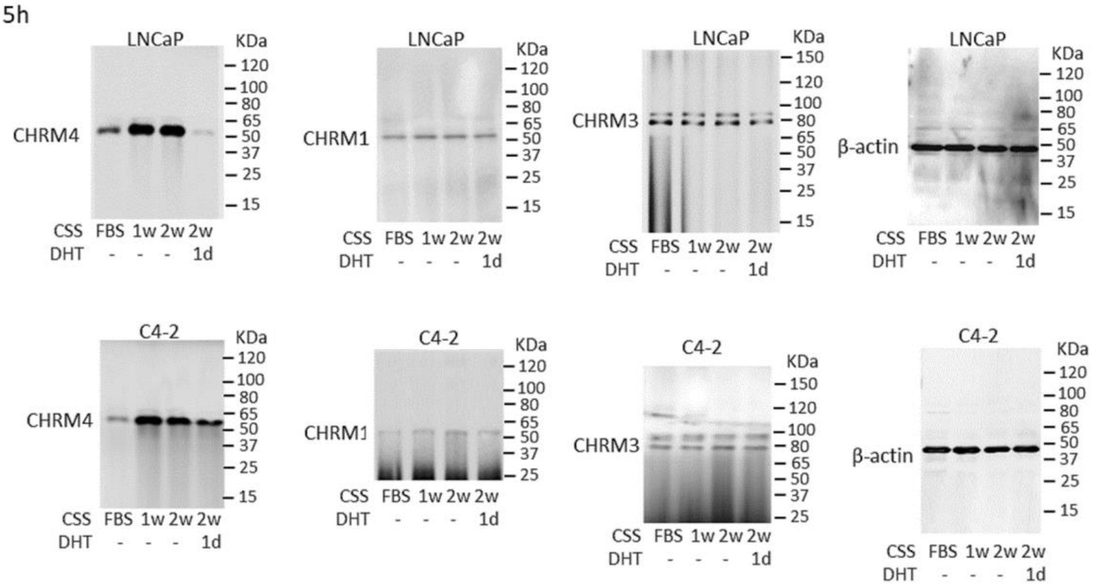
LNCaP
C4-2
KDa
CHRM4
CHRM1
CHRM3
β-actin
150
120
100
80
65
50
37
25
15
CSS FBS 1w 2w 2w
DHT - - - 1d

Fig. 5i

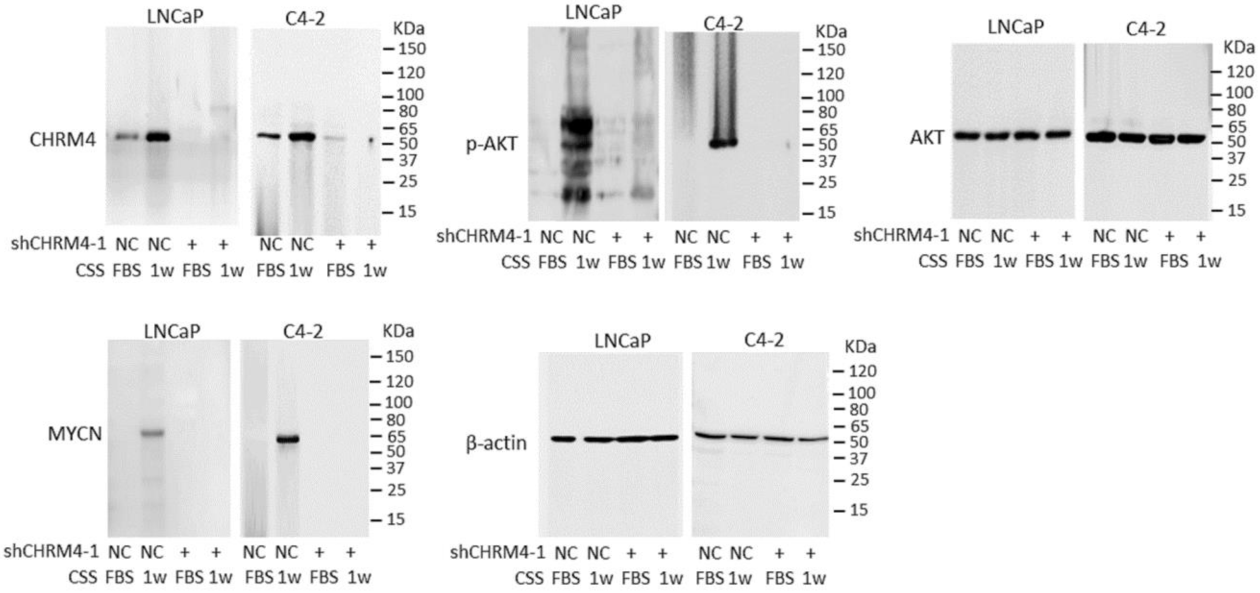
LNCaP
C4-2
KDa
CHRM4
p-AKT
AKT
MYCN
β-actin
150
120
100
80
65
50
37
25
15
shCHRM4-1 NC NC + + NC NC + +
CSS FBS 1w FBS 1w FBS 1w FBS 1w

Fig. 7a

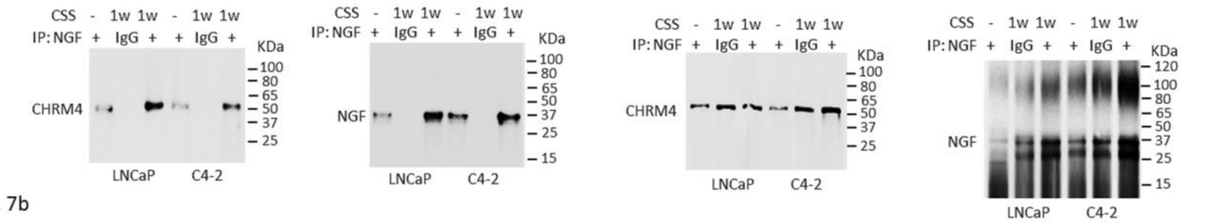

Fig. 7b

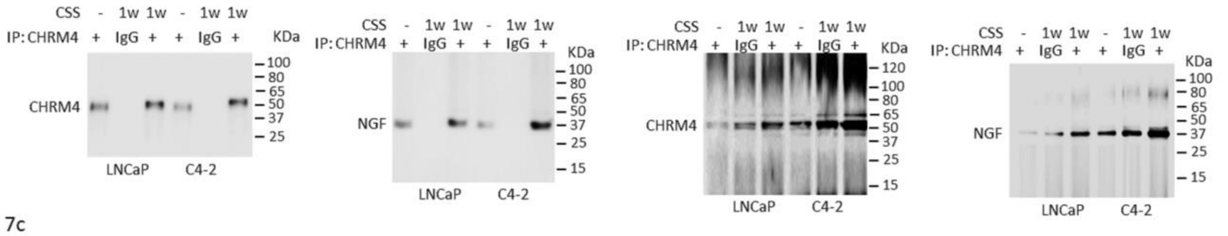

Fig. 7c

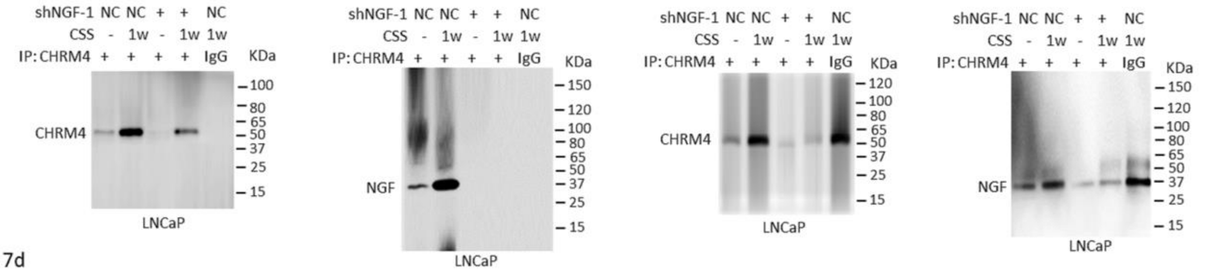

Fig. 7d

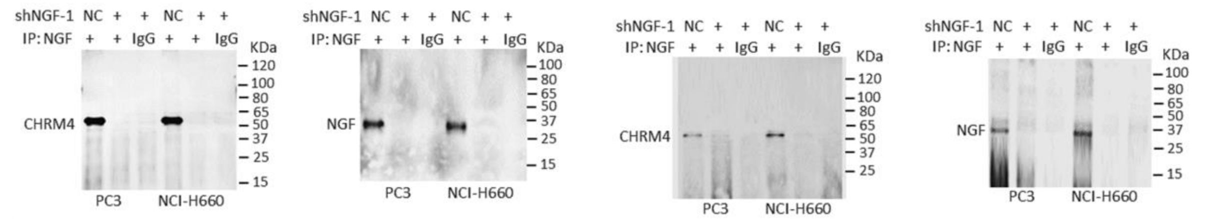

Fig. 7e

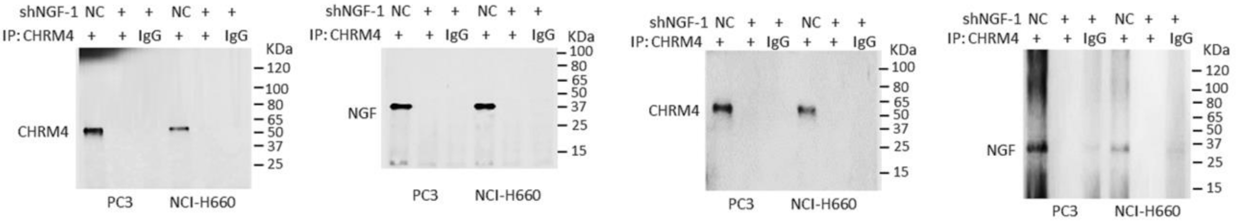

Fig. 7f

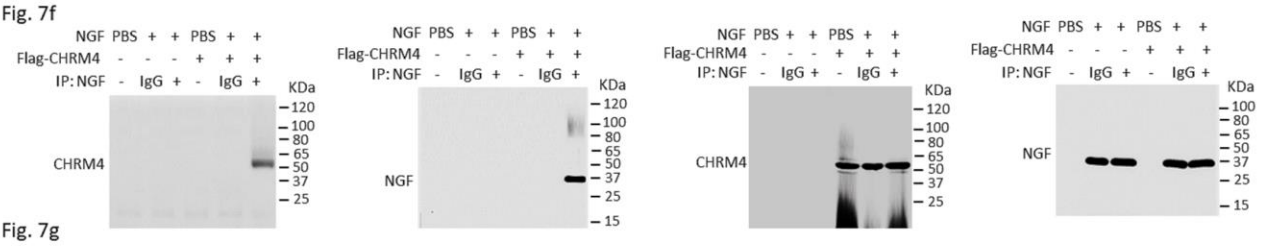

Fig. 7g

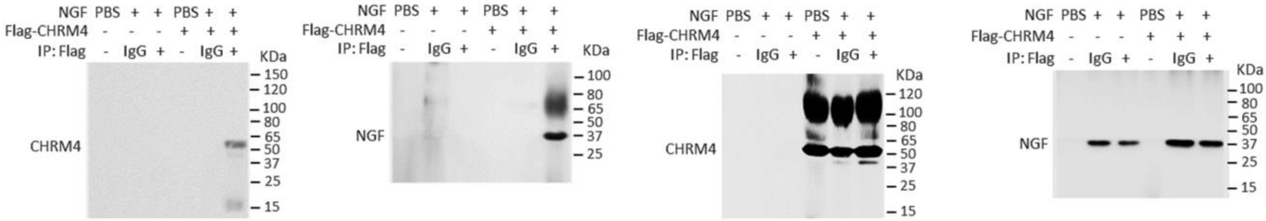

Supplementary tables

Supplementary table 1. Primer sequences of promoter reporter constructs. Primer sequences of the human *NGF* promoter reporter constructs are listed from 5'-3'. (NGF, nerve growth factor; WT, wild-type; M, mutant; F, forward; R, reverse)

Human *NGF* promoter ZBTB46-binding element reporter constructs	
*NGF*_ZEB WT F	ATCCATGCCTTCCTCGACCT
*NGF*_ZEB WT R	ATGTTGCGTACGGGAAACTG
*NGF*_ZEB M F	ATCCAGCAGTATCTCCTACCAACGTTTGCCCCACCAGTT
*NGF*_ZEB M R	GGTAGGAGATACTGCTGGATCAGTCACTGGGA

Supplementary table 2. Real-time RT-PCR primer sequences. Sequences of primers used in the real-time RT-PCR assays in this study are listed 5'-3'. F, forward; R, reverse.

Gene	5'-3'
ZBTB46 F	TCCCTGCTGTTCGAGTACCT
ZBTB46 R	CATGTGTCGCTTGAGGATG
NGF F	CCTTCAACAGGACTCAC
NGF R	CTCCCAACACCATCACCTCC
ENO2 F	GCAGTGGACCACATCAACTC
ENO2 R	TCCAACTCCAGCATCAGGTT
CHGA F	ACTGAAGGAGCTCCAAGACC
CHGA R	TCTGCCTCCTTGGAATCCTC
CHGB F	GCCACGTGCCTATTTCATGT
CHGB R	GCTCCTTCCTCACCGTAGTT
KLK3 F	TTTCCAATGACGTCTGTGCG
KLK3 R	CCAGAATCACCCGAGCAGG
NKX3.1 F	CAGAGACCGAGCCAGAAACG
NKX3.1 R	CTGAGTGTGGGAGAAGGCAG
CHRM4 F	TGGCCACCTACATCTTCCTG
CHRM4 R	CTAGCCACATGAAGGCGAAC
CHRM1 F	CCGACTAAGAAAGGGCGTGA
CHRM1 R	TACGGTGTCCAGGTGAGGAT
CHRM3 F	AGCAAAGCATGAAACGCTCC

CHRM3 R	CTCTCGTCTCGGAGCCAATG
DPYSL5 F	AATGCCTTCATGGTGTGGTC
DPYSL5 R	GCTTAGCCTCGTCGATGAAC
TLX1 F	AGGTTCACAGGTCACCCCTAT
TLX1 R	CAGGTACTTCTGGCGGTGG
FAM148C F	ATCGTGAAGAACAGCCGCC
FAM148C R	TGCCCTTGTTCACCACCTTG
LHFPL5 F	CCATCCTCAGCATTGGCGA
LHFPL5 R	CACACCTCCTCGGTTCCATC
HIST3H3 F	TAAGACCGACCTGCGCTTC
HIST3H3 R	ATGATGGTGACCCGTTTGG
CPLX2 F	CGGCAGCAGATCCGAGATAAG
CPLX2 R	GTATTTGAGCACCGTGTCCAGG
SYP F	CTGTGACCTCGGGACTCAAC
SYP R	CATAGTCAGGCTGGTAGC
PCDH24 F	TACAACACTGAGCGAGCCAA
PCDH24 R	CACAGAGTTGTCGTCCAGG
KIF18B F	CTTTCTTTCCACCAGCCAGC
KIF18B R	GGTCCTGCTGCTTCACAAAG
AKT1 F	GGACAAGGACGGGCACATTA
AKT1 R	CGACCGCACATCATCTCGTA
MYCN F	ACAGTCATCTGTCTGGACGC
MYCN R	TGTCCTCGGATGGCTACAGT
NGFR F	GTATTCCGACGAGGCCAACC
NGFR R	CAGGGATCTCCTCGCACTC
NTRK1 F	GGACCCCATCCCTGACACTA
NTRK1 R	AGGAAGAGGCAGGCAAAGAC
AR F	TCTTGTCGTCTTCGGAAATG
AR R	TCTGGGTTGTCTCCTCAGTG
GAPDH F	CCAGTAGAGGCAGGGATGAT
GAPDH R	CTTTCATTGTCTTTTCCGCC

Supplementary table 3. Western blot antibodies. The source and dilution of each antibody used for Western blotting in this study are listed. cat. no., catalog number.

Primary antibody	Clonality	Source (cat. no.)	Dilution	Secondary antibody	Source	Dilution
ZBTB46	Rabbit polyclonal	GeneTex (GTX121617)	1/1000	anti-rabbit IgG	Jackson Labs	1/5000
NGF	Rabbit monoclonal	Abcam (ab52918)	1/1000	anti-rabbit IgG	Jackson Labs	1/5000
ENO2	Mouse monoclonal	Abcam (ab218388)	1/1000	anti-mouse IgG	Jackson Labs	1/5000
CHGA	Rabbit polyclonal	Abcam (ab68271)	1/1000	anti-rabbit IgG	Jackson Labs	1/5000
KLK3	Mouse monoclonal	Santa Cruz (SC7316)	1/1000	anti-mouse IgG	Jackson Labs	1/5000
NKX3.1	Rabbit monoclonal	Cell Signaling (92998)	1/1000	anti-rabbit IgG	Jackson Labs	1/5000
CHRM4	Rabbit polyclonal	Sigma-Aldrich (SAB4300824)	1/1000	anti-rabbit IgG	Jackson Labs	1/5000
CHRM1	mouse mono clonal	Santa Cruz (SC-265966)	1/500	anti-mouse IgG	Jackson Labs	1/5000
CHRM3	Rabbit polyclonal	Thermo-Fisher (PA5-77485)	1/500	anti-rabbit IgG	Jackson Labs	1/5000
MYCN	Rabbit polyclonal	Cell Signaling (9405)	1/1000	anti-rabbit IgG	Jackson Labs	1/5000
p-AKT	Rabbit polyclonal	Cell Signaling (9271)	1/1000	anti-rabbit IgG	Jackson Labs	1/5000
AKT	Mouse monoclonal	Cell Signaling (4685)	1/1000	anti-mouse IgG	Jackson Labs	1/5000
β-actin	Rabbit polyclonal	GeneTex (GTX109639)	1/10000	anti-rabbit IgG	Jackson Labs	1/20000

Supplementary table 4. ChIP antibodies and primer sequences. The source and dilution of each antibody and the sequences (5’-3’) of each primer used for ChIP in this study are listed. F, forward; R, reverse.

ChIP antibodies				
Primary antibody	Species	Clonality	Source (cat. no.)	Dilution
ZBTB46	Mouse	Monoclonal	Santa Cruz (sc-390260)	1/50
H3K4me3	Rabbit	Polyclonal	Millipore (07-473)	1/50
GAPDH	Rabbit	Polyclonal	Abcam (ab9486)	1/100
Immunoglobulin G	Rabbit		Santa Cruz (sc-2027)	1/50
Immunoglobulin G	Mouse		Santa Cruz (sc-2343)	1/50
ChIP primers				
Site	5'-3'			
*NGF*_ZBE F	ATCCATGCCTTCCTCGACCT			
*NGF*_ZEB R	ATGTTGCGTACGGGAAACTG			
*NGF*_Non-ZEB F	GAGAGGCCAGAGTTGAAACCA			
*NGF*_Non-ZEB R	CAACTCAGTCCTGGGGCTTT			

Supplementary table 5. IHC staining antibodies. The source and dilution of each antibody as well as the method of antigen retrieval used for the IHC assays in this study are listed. HRP, horseradish peroxidase-conjugated. cat. no., catalog number.

Primary antibody	Clonality	Source (cat. no.)	Dilution	Antigen retrieval	Secondary antibody	Source (cat. no.)
ZBTB46	Rabbit polyclonal	Sigma-Aldrich (HPA013997)	1/200	Autoclave	HRP anti-rabbit	DAKO E0432
NGF	Rabbit polyclonal	Abcam (ab6199)	1/100	Autoclave	HRP anti-rabbit	DAKO E0432
CHRM4	Mouse polyclonal	Abcam (ab77956)	1/100	Autoclave	HRP anti-mouse	DAKO E0433
CHGA	Rabbit polyclonal	Abcam (ab15160)	1/100	Autoclave	HRP anti-rabbit	DAKO E0432
ENO2	Mouse monoclonal	Abcam (ab218388)	1/100	Autoclave	HRP anti-mouse	DAKO E0433

Ki67	Rabbit polyclonal	Abcam (ab15580)	1/100	Autoclave	HRP anti-rabbit	DAKO E0432
Cleaved caspase-3	Rabbit polyclonal	Cell Signaling (#9661)	1/100	Autoclave	HRP anti-rabbit	DAKO E0432

Supplementary references

1. Beltran H*, et al.* Divergent clonal evolution of castration-resistant neuroendocrine prostate cancer. *Nat Med* **22**, 298-305 (2016).
2. Nelson PS*, et al.* The program of androgen-responsive genes in neoplastic prostate epithelium. *Proc Natl Acad Sci U S A* **99**, 11890-11895 (2002).
3. Wang G, Jones SJ, Marra MA, Sadar MD. Identification of genes targeted by the androgen and PKA signaling pathways in prostate cancer cells. *Oncogene* **25**, 7311-7323 (2006).
4. Doane AS*, et al.* An estrogen receptor-negative breast cancer subset characterized by a hormonally regulated transcriptional program and response to androgen. *Oncogene* **25**, 3994-4008 (2006).
5. Majumder PK*, et al.* mTOR inhibition reverses Akt-dependent prostate intraepithelial neoplasia through regulation of apoptotic and HIF-1-dependent pathways. *Nat Med* **10**, 594-601 (2004).
6. Kim YH*, et al.* Combined microarray analysis of small cell lung cancer reveals altered apoptotic balance and distinct expression signatures of MYC family gene amplification. *Oncogene* **25**, 130-138 (2006).
