# Supplementary material for: Nerve growth factor interacts with CHRM4 and promotes neuroendocrine differentiation of prostate cancer and castration resistance
Source: Commun Biol. 2021 Jan 4;4:22. doi: 10.1038/s42003-020-01549-1 (PMC7782543; doi:10.1038/s42003-020-01549-1)
Supplement: Supplementary file 2 — Description of Supplementary Files [file 42003_2020_1549_MOESM2_ESM.pdf]

Description of Additional Supplementary Files

File name: Supplementary data 1.
Description: A list of candidate genes from Fig. 5a that were upregulated with an NEPC-responsive gene signature and positively associated with both higher NGF and ZBTB46 expression.

File name: Supplementary data 2.
Description: The source data underlying the graphs and charts presented in the main figures.
